# The Melamine-Driven Solvation Effect Promotes Oxygen Reduction on a Platinum Catalyst: Machine Learning-Aided Free Energy Calculations

**DOI:** 10.1021/acs.jpclett.4c03437

**Published:** 2024-12-24

**Authors:** Ryosuke Jinnouchi, Saori Minami

**Affiliations:** Toyota Central R&D Laboratories, Inc., Nagakute 480-1192, Aichi, Japan

## Abstract

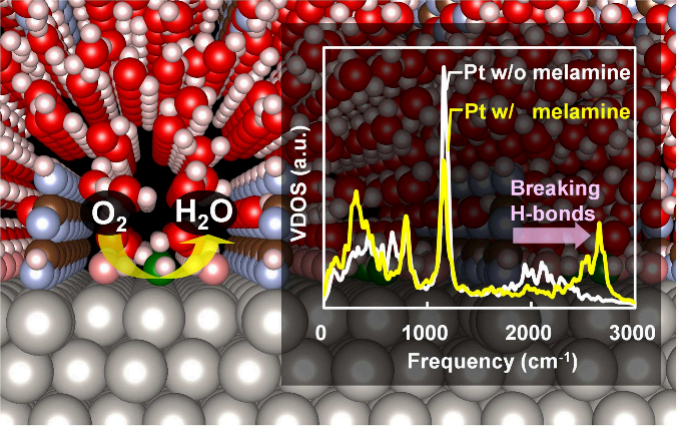

The modification of Pt surfaces with organic compounds like melamine enhances oxygen reduction reaction activity and catalyst durability. Through first-principles free energy calculations utilizing thermodynamic integration and finite-temperature molecular dynamics, enhanced by machine learning force fields for efficient sampling of nanosecond-scale interfacial water fluctuations and incorporating corrections to accurately reproduce first-principles free energies, we demonstrate that melamine destabilizes OH adsorbates, facilitating their removal and enhancing catalytic activity. Unlike alloys, where OH destabilization is driven by changes in electronic structure and surface strain, melamine disrupts hydrogen bonding between OH and interfacial water. Structural and vibrational analyses reveal that melamine alters the water solvation structure, which is evident in modified radial distribution functions and a blue shift in the O–H stretching vibrations. These findings indicate that manipulating interfacial solvation with organic compounds could be a promising approach to enhance catalytic activity without compromising durability.

In the coming decades, achieving green energy and a circular economy will require humanity to shift from fossil fuels to sustainable energy sources that do not emit greenhouse gases. A crucial element in achieving this paradigm shift is the use of electrochemistry to convert electrical energy into hydrogen and to convert hydrogen back into energy without direct combustion in air. Proton exchange membrane fuel cell (PEMFC) is a promising electrochemical system for efficiently converting hydrogen into electricity, both in stationary applications such as homes and buildings, and in mobility applications such as passenger-owned vehicles and heavy-duty vehicles.^1−3^ Intensive research and development efforts have led to remarkable improvements in efficiency and durability. However, for wider adoption, further enhancements in the activity and durability of cathode catalysts are necessary.^4^ According to the New Energy and Industrial Technology Development Organization (NEDO) in Japan, it is estimated that by 2035, catalytic activity must increase more than 9-fold, from 500 A g^–1^ to 4630 A g^–1^. In addition, it is necessary to suppress catalyst dissolution, which leads to degradation through Ostwald ripening during operation.^5,6^

Cathode catalyst needs to activate the oxygen reduction reaction (ORR):
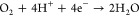
1Platinum (Pt) is the most active single-element catalyst known to date. However, Pt is expensive and scarce, so the amount of Pt used must be reduced. Additionally, to achieve the high efficiency and power density required in mobility applications, it is necessary to further increase catalytic activity while maintaining durability. Among the various methods reported to achieve high activity, alloying Pt with Co or Ni is considered the most established approach. The high activation of Pt through alloying was originally discovered in the research and development of phosphoric acid fuel cells (PAFCs).^7−9^ Subsequently, there has been fundamental and applied researches and developments to utilize similar alloys in PEMFCs.^10−15^ These studies have revealed that monolayer Pt skins on Pt alloys enhances activity. As a result of active research, PtCo nanoparticle alloy catalysts were commercialized in the first and second generations of the Toyota MIRAI.^1,3^

First-principles (FP) calculations have greatly contributed to revealing the mechanism of enhanced activity. The theory and modeling of ORR began with the reproduction of the so-called volcano plot of ORR by No̷rskov and co-workers.^16−20^ In series of these studies, ORR was assumed to proceed mainly through the following associative reaction mechanism according to the past experimental studies:^21^
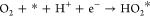
2
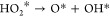
3
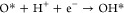
4
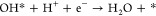
5Here, * denotes the surface site, and X* denotes the species X adsorbed on the surface. The free energies of three reaction intermediates, HO_2_*, OH* and O*, were evaluated using quasi-stable structures at the interface between the catalyst surface and water modeled by hexagonal ice-like bilayers. The computed free energies were then used to calculate the rate constants of catalytic reactions. Since the overall reaction rate is balanced by the formation and removal rates of the intermediates according to the Sabatier principle, a volcano plot emerged when the overall rate was plotted against a property indicating the stability of intermediates, such as the redox potential of the OH* reduction reaction 5. The result indicates the existence of an optimal stability of intermediates for catalytic activity. This calculation result matched well with experimental results, showing that Pt is the optimal catalyst among pure metals. The model was also applied to Pt alloy catalysts and excellently reproduced the experimentally observed volcano plot for monolayer Pt skins on Pt alloy catalysts.^13^ The model also showed that ORR on pure Pt is limited by the removal of OH* (reaction 5) on the catalyst surface, and alloying accelerates this removal step by destabilizing OH*. Combining FP calculations with X-ray analyses^13,14^ also revealed the significant roles of the d-band structure and interatomic distance of surface Pt atoms. This knowledge paved the way for developing a series of core–shell catalysts,^22,23^ dealloyed catalysts,^24^ and shape-controlled alloyed nanoparticles.^25−28^ Furthermore, the FP-based model was applied to search for unknown active catalysts, successfully discovering new Pt alloys with early transition metal elements.^29,30^ It is noteworthy that this methodology has become a foundational method for predicting the activities of a wide variety of catalytic reactions, such as the hydrogen evolution reaction,^31,32^ oxygen evolution reaction,^33,34^ CO_2_ reduction reaction,^35−37^ N_2_ reduction,^38,39^ and more.

However, Pt alloys have limitations. The secondary elements currently introduced into Pt are unstable in acidic electrolytes.^40^ They dissolve during operation, leading to a decrease in catalytic activity due to dealloying. Furthermore, the dissolved elements not only reduce activity but also bind to sulfonic acid groups in the proton exchange membrane, lowering its proton conductivity.^41^ Due to these adverse effects, even though introducing more secondary elements could enhance catalytic activity, it becomes necessary to limit the amount to ensure durability. Despite extensive research to balance activity and durability, very few materials have been found that escape this trade-off relationship.

Recently, it has been found that the catalytic activity can be significantly enhanced by adding organic compounds to the platinum surface.^42−50^ A series of discoveries began with the groups of Sung and Miyabayashi,^42−44^ who found that the addition of amine-terminated organic molecules, such as octylamine, oleylamine, aniline, and hexamethylenediamine, increases the activity of Pt nanoparticle catalysts. Subsequently, Asahi et al.^46^ discovered that melamine, which contains more amino groups in a single molecule, further enhances the activity of Pt nanoparticles. Wada and co-workers^47^ also found the same effect on the Pt(111) single crystal surface, and recently, Hoshi et al.^50^ discovered that adding caffeine to the Pt(111) surface also enhances catalytic activity. Furthermore, Daimon and co-workers^49^ found that melamine also improves the durability of the catalyst. The series of discoveries indicates the presence of a new strategy to enhance catalytic activity without sacrificing durability.

However, the mechanism remains unclear. Both on nanoparticle catalysts and single-crystal electrodes, cyclic voltammetry has shown that organic modification reduces the amount of OH* formed similarly to alloying.^42,47,49,50^ However, it is not yet clear whether this reduction in OH* is simply due to an increase in inactive sites where the surface is covered by organic molecules, preventing the adsorption of oxygen and hydroxides, or whether the organic molecules alter the properties of nearby active sites. Even if organic molecules do change the properties of surrounding active sites, the mechanism remains unknown. Sung’s group argued that the adsorption of organic molecules changes the d-band structure of neighboring surface Pt atoms, destabilizing OH and enhancing activity.^42^ On the other hand, Daimon, Wada, and Hoshi proposed that the presence of organic molecules disrupts the hydrogen bond network, leading to OH destabilization.^47,49,50^ One of the reasons that elucidating the mechanism is difficult is that the surface sites modified by organic molecules are heterogeneous, making it challenging to experimentally determine whether OH* on the active site is destabilized or not. Likewise, experimentally capturing the electronic structure of such atomically heterogeneous interfaces is not straightforward. Furthermore, the challenges in applying FP calculations, which have significantly contributed to understanding alloy effects, also hinder the elucidation of this phenomenon. On surfaces with adsorbed organic molecules, modeling interfacial water in the same way as the ice-like bilayer model on pristine alloys is difficult. For example, on surfaces such as pristine (111), which have good structural alignment with ice, quasi-stable structures obtained at 0 K can be more stable than other configurations, potentially representing the average structure at finite temperatures. This property is one of major reasons FP calculations have been successful in advancing the understanding of alloy effects. However, on surfaces modified by organic molecules, as in this study, the interfacial water structure is significantly disrupted, resulting in a variety of quasi-stable structures within a narrow energy range. Under such circumstances, it becomes difficult to identify a stable structure, and the validity of describing solvent dynamics near such interfaces using harmonic oscillations around a quasi-stable structure at 0 K comes into question. Alternatively, if the dominant quasi-stable structures are very few, and as in previous calculations of solvent effects on high-index surfaces, careful selection of these structures could potentially yield solvent effects consistent with experimental results.^51,52^ However, even in such cases, it is fundamentally essential to present the statistical uncertainties of the calculated averages to determine whether the selected few structures accurately represent the average structure. To evaluate the uncertainties associated with solvent fluctuations at finite temperatures, FP molecular dynamics (MD) would be preferable. However, performing nanosecond-scale simulations required to adequately sample the reorientation of interfacial water^19,53^ demands an enormous computational cost.

Here, we solve the problem by using machine learning (ML)-aided FP calculations of free energies.^59−63^ This approach allows for free energy calculations of OH*, taking into account the fluctuations of water molecules, by employing thermodynamic integration (TI) with finite-temperature MD simulations that are accelerated by several orders of magnitude using machine-learned force fields (MLFFs). Errors in the MLFFs can be efficiently corrected by additional TI calculations from the MLFFs to the FP potential energy surfaces, resulting in precise FP free energies. Using these computed free energies, we calculate the surface redox potentials for reducing OH* to water on single-crystal Pt and Pt alloy (111) surfaces, as well as on the Pt(111) surface with melamine molecules. We also evaluate the catalytic activity using a kinetic ORR model with the computed redox potentials as inputs. As demonstrated by the volcano-plot of catalytic activity versus the redox potential of the OH* reduction (5) shown in Figure 1, our method reproduces the trend of high activity observed experimentally on the melamine-modified Pt surface. By comparing the activities, redox potentials, and the electronic and solvent structures, we propose the mechanism of activity enhancement due to the addition of melamine molecules. Another objective of this study is to establish a method for predicting the free energy of reaction intermediates in catalytic reactions at solid–liquid interfaces, relative to the absolute reference of a noninteracting ideal gas, using TI aided by ML. While MLFFs offer fast computation, they can result in large errors and physically meaningless outcomes in extrapolated regions beyond the training data. In this study, we demonstrate a method that leverages the computational speed of MLFFs while ensuring stable TI within the interpolated space of the training data, thereby enabling FP evaluation of interfacial free energy changes at finite temperatures.

**Figure 1:**
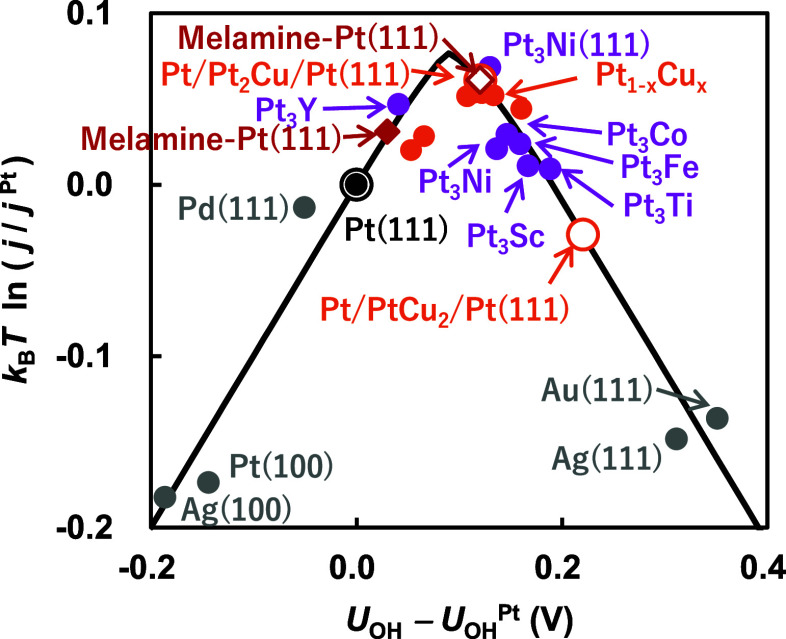
Experimental and simulated volcano plot of the ORR at an electrode potential of 0.9 V vs SHE on Pt(111) (black), Pt(111) with melamine (red), Pt_1–*x*_Cu_*x*_(111) (orange), other Pt alloys (purple), and transition metals (gray) calculated in this study (empty dots and solid black line) and experimental data taken from previous studies (filled dots).^13,14,19,20,29,47,54−58^ Here, the relative change in ORR current density *j* compared to that on Pt(111) is plotted as a function of the redox potential of reaction 5 relative to Pt(111) (*U*_OH_ – *U*_OH_^Pt^). Details of the model in Section S1 of the Supporting Information.

Here, we briefly explain how the thermodynamic stability of OH*, which is the governing factor of ORR activity shown in Figure 1, specifically the redox potential *U*_OH_ of reaction 5, is calculated using the ML-aided TI method. We also describe how the changes in OH* stability due to melamine modification or alloying of Pt appear in the TI calculations. By applying the first-order approximation of interfacial free energy with respect to the excess surface charge proposed by No̷rskov and co-workers,^16,65^ the redox potential *U*_OH_ is approximately expressed as follows (see Section S3 of the Supporting Information for the derivation and discussion of this approximation):
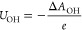
6where Δ*A*_OH_ represents the free energy change due to the reaction:
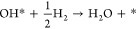
7Δ*A*_OH_ can be written as a sum of two free energy changes:
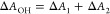
8Δ*A*_1_ represents the free energy change associated with the insertion of a hydrogen atom into the liquid–solid interface,
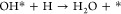
9and Δ*A*_2_ represents the free energy change of the hydrogen dissociation in gas phase,
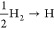
10Δ*A*_2_ can be easily calculated as 2.029 eV at the standard state using the FP method with the revised Perdew–Burke–Ernzerhof semilocal functional,^66^ augmented with Grimme’s dispersion interaction (RPBE+D3),^67,68^ and the ideal gas model. However, Δ*A*_1_ cannot be represented by simple statistical models and requires computationally intensive numerical calculations of statistical averages over phase space for interfacial water, which takes nanoseconds to reorient.^19,53^ To accurately obtain Δ*A*_1_, we use the MLFF-assisted TI calculations from a noninteracting hydrogen atom to an interacting one at the interface:
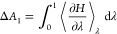
11Here, ⟨.⟩_λ_ means evaluation of the expectation value using an ensemble created by the Hamiltonian at coupling constant λ. This integral seamlessly connects the hydrogen atom in the vacuum (λ = 0) to the one at the interface (λ = 1) along a coupling path. The TI is performed by the modified λ-MLFF scheme,^59,60,62,63^ which allows for computationally efficient insertion of atoms and molecules. As detailed in Section S4 of the Supporting Information, to ensure a smooth and reproducible thermodynamic pathway between the two end points, the modified λ-MLFF scheme introduces an intermediate model potential (its form is provided in eq S27 of the Supporting Information) that guides the system into the interpolated space of the training data, and divides the TI calculation into two steps: (I) TI from the noninteracting hydrogen and the liquid–solid interface involving OH* to hydrogen constrained to OH* by a model potential, and (II) from the model potential to the full potential represented by the MLFF. Additionally, the small error of the MLFF is efficiently corrected by another TI using picosecond-scale MD simulations from the MLFF to the FP potential energy surface.

Figure 2 presents the integrands of TI (see their definitions in eqs S20, S23, and S24 of the Supporting Information) on Pt, melamine/Pt, and Pt/PtCu_2_/Pt surfaces as functions of the coupling constants λ_I_ and λ_II_ in steps I and II, respectively. All calculations were performed by the Vienna Ab initio Simulation Package (VASP).^69−72^ Snapshots of the interfacial structures during the TI calculations are also presented. In the noninteracting state of TI step I (λ_I_ = 0), the hydrogen atoms, represented by slightly larger pink spheres, diffuse freely within the system. Although these noninteracting hydrogen atoms unrealistically overlap with other atoms, the structure of the remaining interacting atoms is accurately represented by the MLFF because the MLFF in the Step I is designed to ignore the presence of these hydrogen atoms (see details in Section S4 of the Supporting Information). Under these conditions, the integrand becomes quite large as the model potential, which determines the integrand, assigns high values to the overlapping configurations. As λ_I_ increases, the model potential gradually starts to act on the system, and the integrand sharply decreases because the repulsive potential prevents the overlaps. Although dense integration grids over λ_I_ are required in this region, the efficient MLFF makes multiple nanosecond-scale MD simulations feasible. At the end point of TI step I (λ_I_ = 1), the hydrogen atoms are bound to the hydroxyl groups shown as green spheres. Because the model potential is the same across all materials, the integrand at this TI step I is insensitive to the material. In TI step II, as the coupling constant λ_II_ increases, the model potential is replaced by the fully interacting potential, eventually leading to the formation of fully interacting water molecules. In this step, material dependence is observed in the integrand because the MLFF is material-dependent. The integrands for melamine/Pt and Pt/PtCu_2_/Pt are slightly lower than those for Pt, suggesting that the OH adsorbates on melamine/Pt and Pt/PtCu_2_/Pt are less stable than those on Pt.

**Figure 2:**
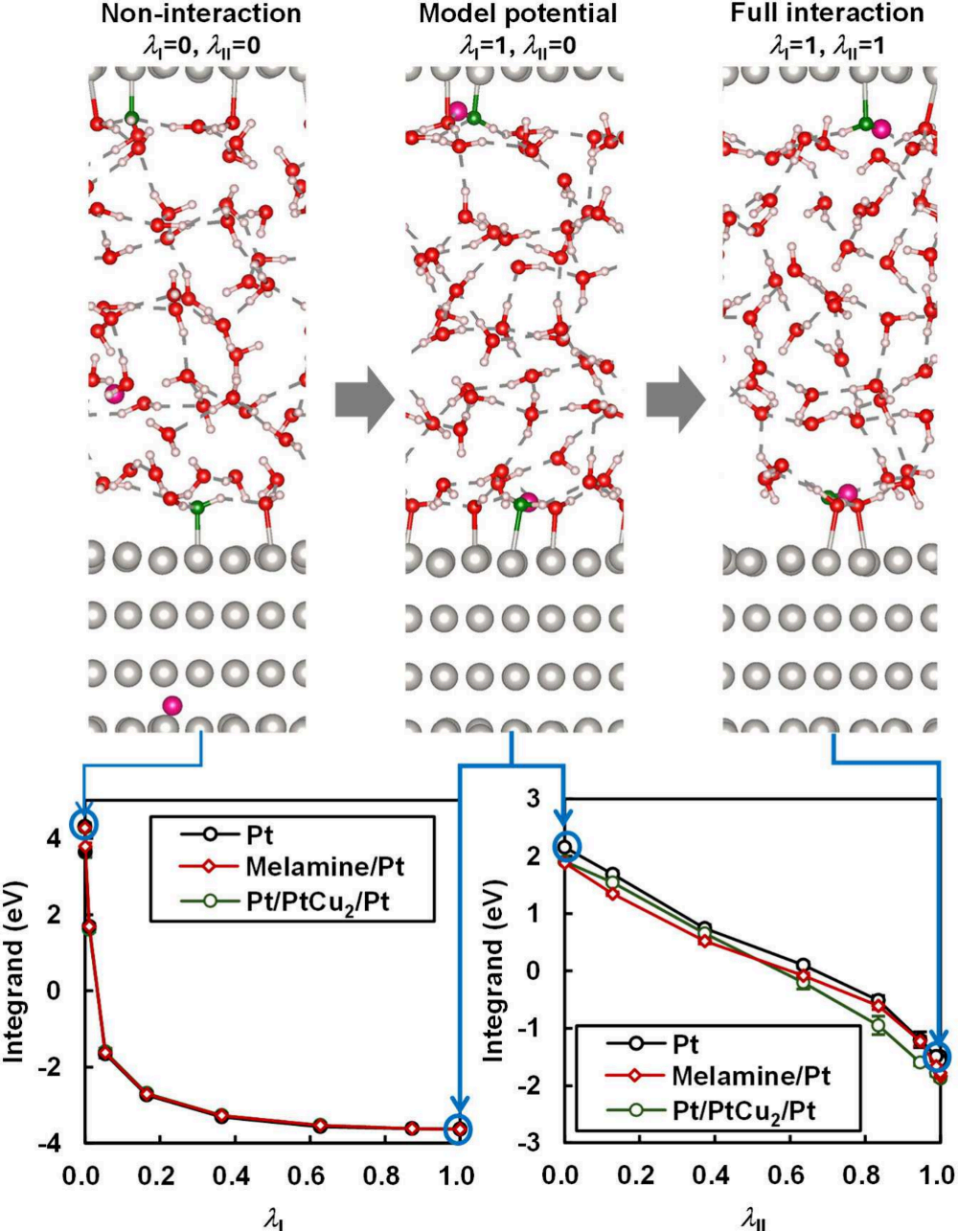
Integrands of the TI calculations in steps I and II (details in Section S4 of the Supporting Information) using the MLFFs on Pt(111), melamine/Pt(111), and Pt/PtCu_2_/Pt(111) surfaces, along with snapshots taken during the TI calculations. Small white spheres represent H atoms, slightly larger spheres in a darker shade of pink the inserted hydrogen atoms, red medium spheres oxygen atoms in H_2_O, green medium spheres oxygen atoms in OH adsorbates, and silver large spheres Pt atoms. The statistical error bars of the integrands, which are too small to be seen, represent the standard deviation determined through block averaging analysis. Graphic images were generated by VESTA.^64^

The redox potentials (*U*_OH_) for the OH* reduction reaction 5 obtained from the TI calculations using the MLFF (via eqs S19 and S20 of the Supporting Information) and the FP method (via eqs S18, S21, and S22 of the Supporting Information) are shown in Figure 3a. A notable point is that all the MLFFs generated in this study were able to reproduce the redox potentials obtained by the FP method with very high accuracy, achieving an RMSE of 50 mV. This result indicates that the MLFF can provide reliable predictions for free energies of solid–liquid interfaces. In the following discussion, we focus on the FP results. For comparisons with the reported experimental result,^58^ the shifts in the redox potential (Δ*U*_OH_ = *U*_OH_ – *U*_OH_^Pt^) on the near-surface alloys Pt/Pt_1–*x*_Cu_*x*_/Pt(111), relative to that on Pt(111), are presented in Figure 3b, as was done in the previous study. In the same figure, the present results are also compared with previous FP results calculated using the harmonic oscillator model and structures optimized at 0 K.^58^ These approximations are considered reasonable for pristine (111) surfaces without melamine, thereby providing a reliable validation of the present method. Similar to the previous experiment and FP calculation, our TI calculation indicates that the redox potential gradually increases with the amount of Cu (*x*) in the second layer from the surface (see the surface models in Figure S6). The calculation, however, overestimates the extent of *U*_OH_ increase compared to the experiment. In addition, at *x* = 1, the results of the present study deviate more significantly from the experimental values compared to the previous method. These discrepancies arise because the elemental distribution in the experiment is not as idealized as in the model, and the atomic composition of the third layer from the surface in the symmetrical slabs used in this study differs from that of the experimental surface and the previous model. For instance, at *x* = 1, the third layer of the current slab consists of a pure Cu layer, as shown in Figure S6e, whereas in the previous slab model, it was a pure Pt layer. However, the primary objective of this study is to compare the effects of alloying and melamine modification, and the differences arising from such model variations do not pose a fundamental issue.

**Figure 3:**
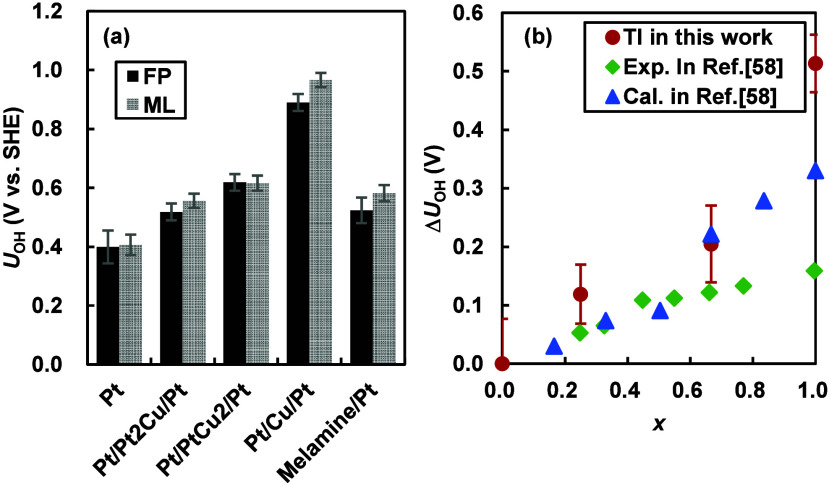
(a) Redox potentials vs SHE for reaction 5 on Pt(111), Pt(111) with melamine, Pt/Pt_2_Cu/Pt(111), Pt/PtCu_2_/Pt(111), and Pt/Cu/Pt(111) surfaces obtained from TI calculations and (b) values on the alloy surfaces relative to those on Pt(111), compared with previous experimental and calculated results.^58^ In panel a, ML represents the MLFF results obtained using eqs S19 and S20 while FP represents the FP results obtained by correcting the ML results with eqs S18, S21, and S22. In panel b, the previous calculations shown were conducted using the conventional FP method^16^ with harmonic oscillator models and structures optimized at 0 K.

Figure 3a also shows the computed redox potential on the melamine-modified Pt(111) surface compared to those on the near-surface alloys. The melamine modification leads to a 0.12 V upward shift of the redox potential, indicating that OH adsorbate is destabilized by melamine. This result is consistent with the experimental cyclic voltammograms, which showed reductions in the amount of OH* on melamine-modified Pt surfaces (see also simulated OH* coverages in Figure S1).^47,49,50^ Experimentally, it was unclear whether this reduction was due to a reduction in hydroxyl adsorption sites caused by melamine or a change in the hydroxyl affinity of the surrounding active sites near melamine. Our simulation clearly demonstrates that the reduction is indeed caused by the latter mechanism.

Using the calculated shift in the redox potential Δ*U*_OH_, the relative change in ORR current density *j* from that on pure Pt(111) was evaluated with the simple kinetic model proposed by Rossmeisl and co-workers (see Section S1).^18,19^ As shown in Figure 1, similar to previous studies, this model reveals a volcano-type correlation between the activity and the redox potential. Notably, in the simulation, the melamine-modified Pt surface is positioned very close to the optimal point on the volcano plot, similar to the Pt/Pt_2_Cu/Pt(111) surface, achieving an order of magnitude higher catalytic activity than Pt(111). This suggests that our calculations show melamine destabilizes the OH adsorbate and accelerates the OH* removal step 5, which is the rate-limiting step on the Pt(111) surface without melamine, thereby enhancing ORR activity. However, the simulation overestimates the impact of melamine on both Δ*U*_OH_ and activity compared to the experimental results. In experiments, it is known that the melamine coverage strongly influences both Δ*U*_OH_ and activity. Although further investigation into the coverage dependence is necessary, this discrepancy is potentially attributable to the higher surface coverage of melamine in the simulation (0.08 ML) compared to the experimental value (0.06 ML).

Both melamine modification and alloying activate ORR by destabilizing OH*. However, the mechanisms by which OH* is destabilized are completely different between the two surfaces. As shown in the projected density of states (PDOS) in Figure S7, the d-band of the Pt atom located directly beneath the melamine is downshifted in energy. This trend qualitatively matches the previously reported shifts in the d-band of surface Pt atoms with organic molecules.^42,73^ However, it is evident that OH cannot adsorb onto the Pt atom directly beneath the melamine. As shown in Figure S7, the changes in the electronic structure caused by melamine are localized. Furthermore, as demonstrated in Figure 4a, the PDOS of the d-band for the surface Pt atom, where OH is stably adsorbed during finite-temperature MD, closely matches that of a surface Pt atom without melamine, indicating that the modification of the d-band by melamine is minimal. The shift in the d-band center of the OH-adsorption site is only 8 meV, which is orders of magnitude smaller than the downshifts of 200 to 300 meV observed in Pt alloys containing Ni and Co in previous studies.^13,14^ The localized nature of the electronic effect of melamine is also reflected in the Bader charge analysis^74^ (see Figure S7). The Pt atom directly beneath the melamine, bonded to the N atoms in melamine, is positively charged due to electron withdrawal by melamine, while the charge on the nearest Pt atom closely matches that of a Pt surface without melamine. These results suggest that the destabilization of OH* is not due to a downshift in the d-band. Furthermore, as shown in Figure 4b, unlike the alloyed Pt surface containing Cu, which was also calculated in this study, melamine does not alter the distance (*d*) between the surface and subsurface layers. On the Pt–Cu alloys, the shortening of the metal–metal distance leads to the destabilization of the OH adsorbate via the strain effect,^29^ but these results indicate that the destabilization caused by melamine is not due to the strain effect. The difference in the mechanism is also evident in the shift in the calculated redox potential (Δ*U*_OH_) relative to the pure Pt(111) surface in vacuum, as shown in Figure 4c. On alloyed surfaces, the positive shift occurs even in a vacuum, whereas on the Pt surface with melamine, the positive shift is observed only in water.

**Figure 4:**
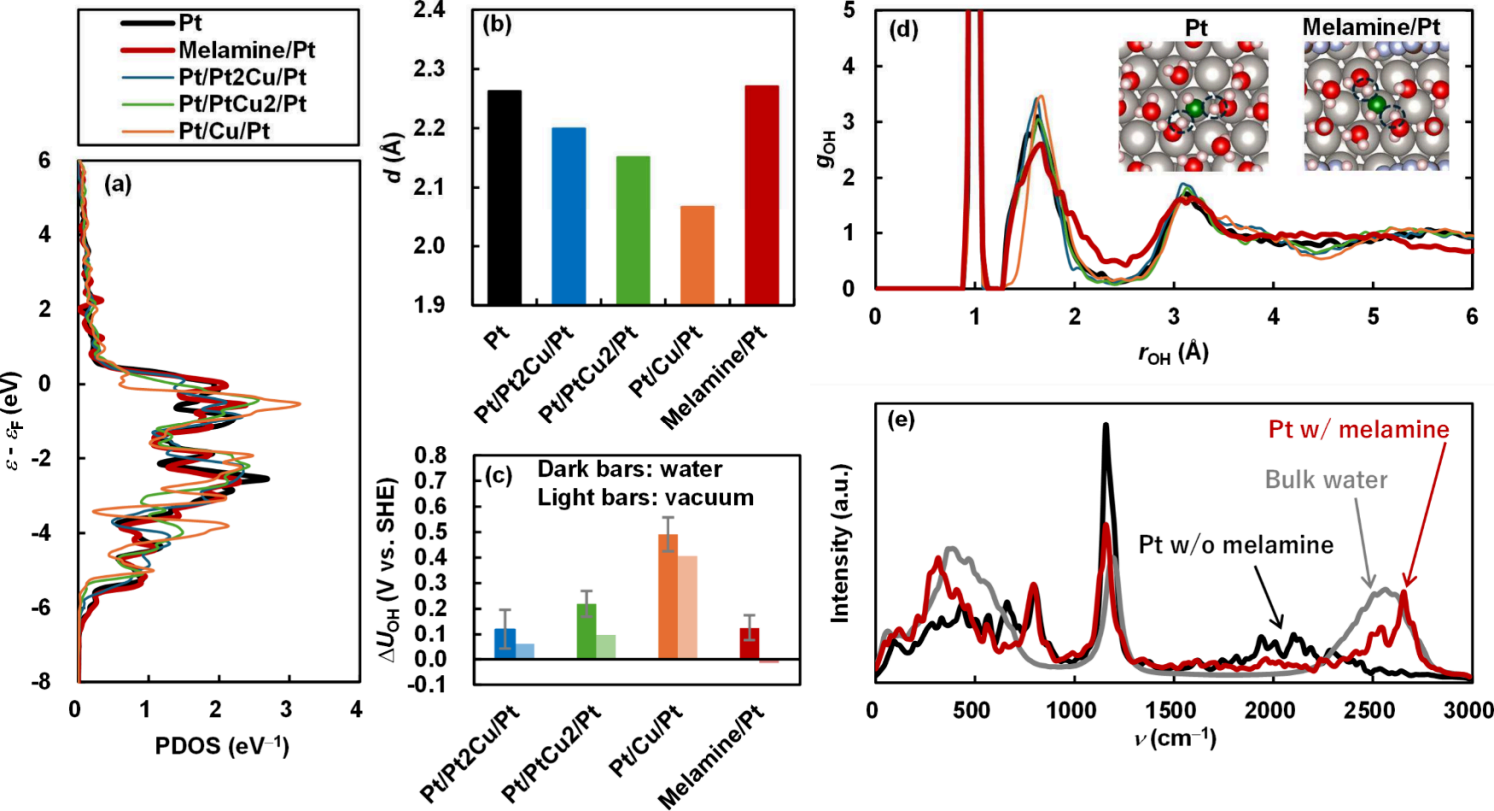
(a) PDOS for the d-band of the surface Pt atom where OH is adsorbed, (b) distance (*d*) between the surface and subsurface layer, (c) redox potentials in water and vacuum relative to the Pt(111) surface, (d) RDF between the oxygen atom in the OH adsorbate and hydrogen atoms in water and interfacial water structures on the Pt surfaces without and with melamine, and (e) vibrational DOS of deuterized hydrogen atoms within the second peak of the RDF, on the Pt(111) (black), Pt(111) with melamine (red), Pt/Pt_2_Cu/Pt(111) (blue), Pt/PtCu_2_/Pt(111) (green), and Pt/Cu/Pt(111) (orange) surfaces. The PDOS and the redox potentials in vacuum were obtained by the FP calculations for the slab models in vacuum expalined in Section S7 of the Supporting Information. The vibrational DOS was obtained by performing Fourier transforms of the velocity autocorrelation functions of hydrogen atoms within the second peak of the RDFs (1.25 Å ≤ *r* ≤ 2.50 Å). These atoms correspond to those enclosed by the dashed circles in the figure insets. The velocity autocorrelation functions were calculated using 10 ps FPMD simulations, which were initiated from the final structures of 1.6 ns MD simulations using MLFFs.

Figure 4c indicates that the destabilization of OH* caused by melamine is thought to result from changes in the solvation structure induced by the adsorbed melamine. As shown in Figure 4d, melamine broadens the second peak at 1.7 Å in the radial distribution function (RDF) between the oxygen atom of the OH adsorbate and the hydrogen atoms of water molecules and reduces the peak height, whereas alloying Pt with Cu does not alter the RDF. This suggests that melamine weakens the hydrogen bonds between water and OH*. Signs of hydrogen bond weakening are also evident in the vibrational density of states (DOS) of deuteralized hydrogen atoms near the OH adsorbate shown in the insets of Figure 4d. On the Pt surface without melamine, the vibrational frequency of the O–D stretching mode exhibits a red shift to approximately 2000 cm^–1^ compared to the bulk liquid water value of 2650 cm^–1^. This significant red shift is attributed to strong hydrogen bonds between interfacial water molecules and the OH adsorbate. In contrast, on the Pt surface with melamine, the O–D stretching band is instead blue-shifted, and the red-shifted band diminishes. This indicates that melamine weakens the hydrogen bonds. The mechanism by which OH* is destabilized and ORR is accelerated due to the disruption of the solvation structure around OH* has been proposed to occur through the introduction of one-dimensional step defects^51,75−78^ or hydrophobic cations^50^ on the Pt(111) crystal surface. The results of this calculation indicate that melamine promotes ORR through the same mechanism. It should be also noted that the blue shift of the interfacial water O–D(H) vibrations, shown in the VDOS, may be experimentally detectable.^79,80^ Such detection would provide critical evidence supporting the validity of the mechanism predicted by our calculations.

In summary, using FP free energy calculations with MD simulations aided by MLFFs, we propose the mechanism by which melamine modification enhances ORR activity on Pt catalysts. Calculations of the OH* reduction potential revealed that, similar to Pt alloying, melamine destabilizes OH* on Pt and accelerates the OH removal process, which is the rate-limiting step of ORR. However, analyses of the electronic structure and surface atomic distances indicated that the mechanism of OH* destabilization by melamine is entirely different from the electronic effects of a d-band center downshift or strain effects caused by shortened metal–metal distances observed in alloys. FPMD analyses of solvent structure and vibrational frequencies demonstrated that melamine destabilizes OH* by disrupting the hydrogen bonding around it, thereby promoting ORR. In other words, modification with organic molecules like melamine achieves high catalytic activity through solvation effects. This advanced approach is expected to offer a new guideline for catalyst design that surpasses the traditional trade-off between activity and durability seen in alloys. In addition, the results of this study demonstrate that FP calculations of redox potentials aided by MLFFs offer a method for predicting free energy changes of elementary reactions at complex solid–liquid interfaces at finite temperatures, without relying on a harmonic oscillator model for structures optimized at 0 K. Currently, MLFFs do not always fully replicate FP results, and the potential errors they may introduce in redox potentials remain unclear. Consequently, computationally intensive TI from MLFFs to FP potentials is required to address this uncertainty, which restricts their application to more rigorous calculations for multiple charged surfaces, beyond the electrically neutral surface approximations applied in this study, as well as applications to large-scale systems. In this study, however, the errors associated with MLFFs were found to be relatively small, approximately 50 mV, and they successfully reproduced the trends observed in FP calculations. As the accuracy of MLFFs continues to improve, they are expected to offer reliable predictions without the need for such corrections. Nonetheless, like any ML models, they may yield erroneous results for structures outside their training data sets. Thus, even in the future, the current approach will remain essential for verifying uncertainties in ML predictions. We believe this method not only offers valuable insights into the effects of organic molecules on ORR, as explored in this study, but also paves the way for FP predictions of a wide range of catalytic reaction mechanisms at electrodes. These include cation effects on ORR, the hydrogen evolution reaction, CO_2_ reduction,^81−84^ and solvation effects on the oxygen evolution reaction (OER) on oxide surfaces.^85^

## Supporting Information Available

Descriptions of the kinetic model for the ORR, formulations and accuracy of the MLFFs, free energy formulations, ML-aided TI schemes, interfacial models, details of the PDOS and Bader charges, free energies in vacuum, adsorption structures of melamine molecules, and potentials of zero charge for Pt surfaces with and without melamine covered by liquid water (PDF)
