# Correction to “The Melamine-Driven Solvation Effect Promotes Oxygen Reduction on Platinum Catalyst: Machine Learning-Aided Free Energy Calculations”

**DOI:** 10.1021/acs.jpclett.5c01222

**Published:** 2025-05-06

**Authors:** Ryosuke Jinnouchi, Saori Minami

In our original manuscript, we inadvertently used base-10 logarithms instead of natural logarithms in implementing eqs (S32) and (S33) of the Supporting Information, which are used to calculate the vibrational quantum correction for the hydrogen atom bonded to water. This error resulted in an overestimation of the free energy of the hydrogen atom by approximately 60 meV, thereby lowering the redox potential *U*_OH_ associated with OH adsorption on Pt catalysts both with and without melamine, as well as on various Pt alloys by 60 mV, shown in Figure 3 (a). A corrected version of Figure 3 (a), along with updated vibrational quantum correction values in Table S3 of the Supporting Information, is provided below. Since the evaluation of catalytic activity in our study relied on relative differences in *U*_OH_ among the Pt catalysts, this uniform shift in absolute values does not alter the other results presented. Consequently, the main conclusions of the study remain unaffected. Nonetheless, the corrected absolute value of *U*_OH_, for instance, on Pt(111) increases from 0.40 to 0.46 V vs SHE, bringing it closer to the experimental reference value of 0.6 V vs SHE in ref. [14] of the original manuscript.

**Figure 3:**
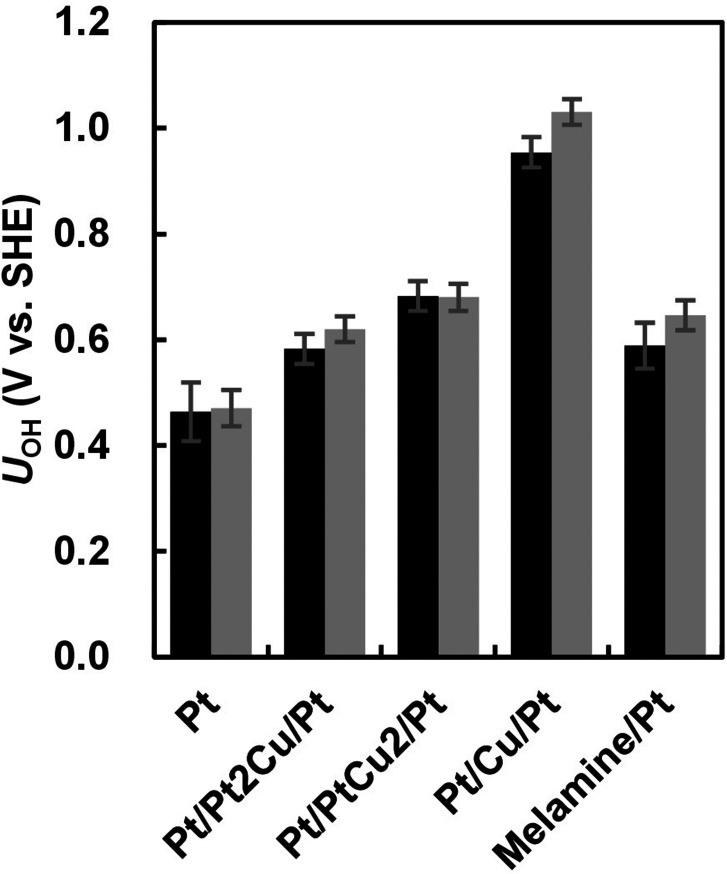
(a) Redox potentials versus SHE for reaction 5 on Pt(111), Pt(111) with melamine, Pt/Pt_2_Cu/Pt(111), Pt/PtCu_2_/Pt(111), and Pt/Cu/Pt(111) surfaces obtained from TI calculations.

**Table S3:** Nuclear quantum effects on the free energies of OH* and H_2_O at the interface between the water bilayer and Pt(111) surface^a^

Property	OH*	H_2_O
ν_*i*_	3571	3594
	958	3503
	785	1621
	389	640
	258	556
	234	541
		199
		152
		53
*A*_q, vib_	**0.360**	**0.601**
*A*_c, vib_	**0.169**	**0.234**
*A*_q–c_	**0.192**	**0.367**

a The units of free energy and vibrational frequencies are eV and cm^–1^, respectively. Corrected values are indicated in bold.

