# Supplementary material for: The Melamine-Driven Solvation Effect Promotes Oxygen Reduction on a Platinum Catalyst: Machine Learning-Aided Free Energy Calculations
Source: J Phys Chem Lett. 2024 Dec 24;16(1):265–73. doi: 10.1021/acs.jpclett.4c03437 (PMC11726801; doi:10.1021/acs.jpclett.4c03437)
Supplement: Supplementary file 1 — jz4c03437_si_001.pdf [file jz4c03437_si_001.pdf]

# Supporting Information for Melamine-Driven Solvation Effect Promotes Oxygen Reduction on Platinum Catalyst: Machine Learning-Aided Free Energy Calculations

Ryosuke Jinnouchi* and Saori Minami

*Toyota Central R & D Labs., Inc., Nagakute 480-1192 Aichi, Japan*

E-mail: jryosuke@mosk.tytlabs.co.jp

## S1. Kinetic model of ORR

The central objective of this study is to calculate the redox potential $U_{\mathrm{OH}}$ of the OH* reduction to water using finite-temperature MD simulations that can account for interfacial solvation effects, with the assistance of MLFFs. However, before explaining the MD method, we begin by explaining how $U_{\mathrm{OH}}$ is related to the catalytic activity of the ORR. To achieve this, we begin with a simple kinetic model of the ORR proposed by Rossmeisl and co-workers,[1] which is well-known for its excellent ability to reproduce the trend in catalytic activity of the ORR across a wide range of materials,[2] using a single property of the catalyst, specifically $U_{\mathrm{OH}}$, as shown in Fig. 1.

This model primarily relies on three assumptions. First, the ORR is assumed to be limited by either the $HO_2$ formation reaction (2) or the OH* removal reaction (5). This assumption has been supported by more refined kinetic models and experiments.[2–5] Second, the contribution of the backward reaction (2) is negligible due to the large overpotential under which the ORR occurs. Third, the catalyst that stabilizes OH* is assumed to stabilize $HO_2$* by the same energetic extent, due to a property known as the scaling relation.[6] This relation has also been validated by FP calculations for various catalysts.[2,5–7] Based on the first and second assumptions, the steady-state ORR current density $j$ can be simply written as

$$j = j_{\mathrm{limit}} \frac{k_2 k_5}{k_2 + k_5 + k_{-5}}, \tag{S1}$$

where $j_{\mathrm{limit}}$ represents the material-independent turnover frequency per site limited by the diffusion of the reactant, $k_2$ is the rate constant of the forward reaction (2), and $k_5$ and $k_{-5}$ are the rate constants of the forward and backward reactions (5), respectively.

Equation. (S1) can be derived as explained below (see also Ref.[1]). The first assumption outlined in the previous paragraph implies that the rates of elementary reactions other than reactions (2) and (5) are fast. This means that the surface coverages of $HO_2$* and O* are

always low. Since the backward reaction of reaction (2) is minimal, the steady-state current density of ORR can be expressed as follows:

$$j = j_{\text{limit}}(1 - \theta_{\text{OH}})k_2, \tag{S2}$$

where $\theta_{\text{OH}}$ is the surface coverage of OH*. Under steady-state conditions, the surface coverage of OH* remains unchanged. Therefore, the following equation holds:

$$\frac{d\theta_{\text{OH}}}{dt} = (1 - \theta_{\text{OH}})\, k_2 - \theta_{\text{OH}} k_5 + (1 - \theta_{\text{OH}})k_{-5} = 0, \tag{S3}$$

Equation (S3) yields the steady state coverage as:

$$\theta_{\text{OH}} = \frac{k_2 + k_{-5}}{k_2 + k_5 + k_{-5}}. \tag{S4}$$

Substituting Eq. (S4) into Eq. (S2) leads to Eq. (S1).

These rate constants are expressed using the Butler-Volmer equation[8,9] as

$$k_{\pm i} = k_i^0 \text{Min}\left[1, \exp\left(\mp \frac{\alpha_i\,(U - U_i)}{k_{\text{B}} T}\right)\right], (i = 2 \text{ or } 5) \tag{S5}$$

where $k_i^0$ represents the prefactor, $\alpha_i$ is the transfer coefficient, $U$ is the electrode potential, $U_i$ is the redox potential ($U_2 = U_{\text{HO}_2}$ and $U_5 = U_{\text{OH}}$), $k_{\text{B}}$ is the Boltzmann constant, and $T$ is the temperature. The prefactor $k_i^0$ depends on the activation barrier for interfacial proton transfers, which can vary depending on the material. However, previous studies[1–5,10] have shown that the ORR is thermodynamically limited by the OH formation and removal steps, making the material-dependence of the reaction rate insensitive to the prefactor. Consequently, in most kinetic ORR models from prior studies, the prefactor is typically assumed to be independent of the material. As demonstrated in Fig. 1 and corroborated by other studies,[1–5,10] this kinetic model consistently describes the ORR activity on (111),

(100), (110), and high-index surfaces, all of which are covered by structurally distinct water layers, in a unified manner. Therefore, we also utilized material-independent constant prefactors in this study. The third assumption of the scaling relation implies the relation $U_{\mathrm{OH}} - U_{\mathrm{OH}}^{\mathrm{Pt}} = -\left(U_{\mathrm{HO_2}} - U_{\mathrm{HO_2}}^{\mathrm{Pt}}\right)$, where $U_{\mathrm{OH}}^{\mathrm{Pt}}$ and $U_{\mathrm{HO_2}}^{\mathrm{Pt}}$ are the redox potentials on the Pt(111) surface.[1,2,5] Consequently, the ORR current density $j$ relative to Pt(111) can be expressed as a function of the single variable $\Delta U_{\mathrm{OH}} = U_{\mathrm{OH}} - U_{\mathrm{OH}}^{\mathrm{Pt}}$. In this study, following the empirical adjustments made by Rossmeisl and co-workers,[1] $k_2^0$ and $k_5^0$ were set to 0.1 and 1.0, respectively. In addition, $U_{\mathrm{OH}}^{\mathrm{Pt}}$ was also adjusted to 0.8 V vs. SHE. The material-dependent variation $\Delta U_{\mathrm{OH}}$ in the redox potential was calculated using the machine-learning-aided FP free energy calculation method described in Sections S3 to S5. The aim of this study is to develop a TI method for predicting $U_{\mathrm{OH}}$ and to elucidate the effects of melamine on $\Delta U_{\mathrm{OH}}$.

As shown in Fig. 1, this model provides results that are in good agreement with experiments, predicting that the relative change in ORR current density $j$ compared to that on Pt(111) exhibits a volcano-type correlation with the change in redox potential $\Delta U_{\mathrm{OH}}$. Catalysts on the left wing of this plot have too low $U_{\mathrm{OH}}$ for the optimal condition due to the excessive stabilization of OH*. Under this condition, $k_2 \gg k_5$, $k_{-5}$, and thus, the reaction is limited by the OH* removal reaction (5) ($j \simeq j_{\mathrm{limit}} k_5$). On the other hand, catalysts on the right wing are limited by the $HO_2$* formation reaction (2) ($j \simeq j_{\mathrm{limit}} k_2$, $k_5 \gg k_2$, $k_{-5}$). The pure Pt(111) surface is positioned on the left wing and has slightly too strong binding energy with OH*, making the OH* removal reaction (5) slightly slower. Alloying subsurface layers causes a downshift in the d-band of the surface Pt atoms and/or compresses the metal-metal distances,[2,4,11–13] which destabilizes OH* and thereby accelerates the ORR. However, excessive addition of a secondary element can destabilize $HO_2$*, slowing down reaction (2) and reducing activity. The model also provides OH* surface coverage in reasonable agreement with experimental observations,[14–17] as demonstrated in Fig. S1 and Ref.[1] On the pristine Pt(111) surface in an inert atmosphere without oxygen gas, the model indicates that OH* formation starts at approximately 0.65 V and reaches around 1/3 ML coverage at

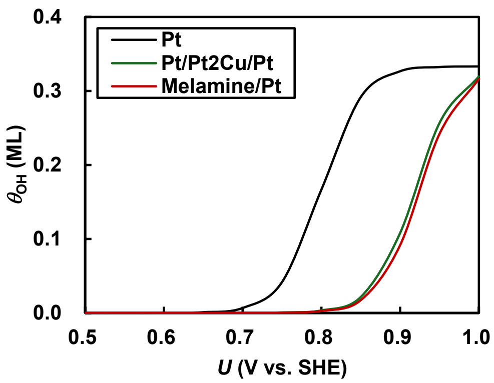


Figure S 1: Simulated surface coverage of OH* on the pure Pt, Pt/$Pt_2$Cu/Pt, and melamine-modified Pt surfaces in an inert atmosphere without oxygen gas using $\Delta U_{\mathrm{OH}}$ calculated by the machine-learning-aided FP free energy calculations. Similarly to Ref.,[1] $\theta_{\mathrm{OH}}$ was rescaled to correspond to 1/3 ML at the fullly covered state.

slightly above 0.9 V as shown in Fig. S1 or in Ref.[1] Furthermore, for the Pt alloy and the melamine-modified Pt surface, whose $\Delta U_{\mathrm{OH}}$ were computed to be positive by the machine-learning-aided FP free energy calculations, the model predicts lower OH* coverages due to the increased redox potential required for OH formation, a trend that is consistent with experimental findings.[16,17]

## S2. Formulations, parameters and accuracy of MLFFs

Similar to previous machine-learning approaches,[18,19] the potential energy $U$ of a structure with $N_{\mathrm{a}}$ atoms in our MLFF method is approximated as a summation of local energies $U_i$ written as

$$U = \sum_{i=1}^{N_{\mathrm{a}}} U_i. \tag{S6}$$

Following the Gaussian approximation potential pioneered by Bártok and co-workers,[19] the local energy $U_i$ is approximated as a weighted sum of functions $K(\mathbf{x}_i, \mathbf{x}_{i_{\mathrm{B}}})$ centered at reference points $\{\mathbf{x}_{i_{\mathrm{B}}} | i_{\mathrm{B}} = 1, ..., N_{\mathrm{B}}\}$

$$U_i = \sum_{i_{\mathrm{B}}=1}^{N_{\mathrm{B}}} w_{i_{\mathrm{B}}} K(\mathbf{x}_i, \mathbf{x}_{i_{\mathrm{B}}}). \tag{S7}$$

The coefficients $\{w_{i_{\mathrm{B}}} | i_{\mathrm{B}} = 1, ..., N_{\mathrm{B}}\}$ are optimized to best reproduce the FP energies, forces, and stress tensor components as obtained by the FPMD simulations. The descriptor $\mathbf{x}_i$ used in this study is a vector containing two and three body contributions:[20]

$$\mathbf{x}_i^{\mathrm{T}} \rightarrow \left( \sqrt{\beta^{(2)}} \mathbf{x}_i^{(2)\mathrm{T}}, \sqrt{\beta^{(3)}} \mathbf{x}_i^{(3)\mathrm{T}} \right), \tag{S8}$$

Here, $\beta^{(2)}$ and $\beta^{(3)} (= 1 - \beta^{(2)})$ are the weights on the two and three body descriptors, $\mathbf{x}_i^{(2)}$ and $\mathbf{x}_i^{(3)}$, respectively. The vectors $\mathbf{x}_i^{(2)}$ and $\mathbf{x}_i^{(3)}$ collect the expansion coefficients of two and three body distribution functions with respect to the orthonormal radial and angular basis sets:[20,21]

$$\rho_i^{(2)}(r) = \frac{1}{\sqrt{4\pi}} \sum_{n=1}^{N_{\mathrm{R}}^0} c_n^i \chi_{n0}(r) \tag{S9}$$

$$\rho_i^{(3)}(r, s, \theta) = \sum_{l=0}^{L_{\max}} \sum_{n=1}^{N_{\mathrm{R}}^l} \sum_{\nu=1}^{N_{\mathrm{R}}^l} \sqrt{\frac{2l+1}{2}} p_{n\nu l}^i \chi_{nl}(r) \chi_{\nu l}(s) P_l(\cos\theta). \tag{S10}$$

The two and three body distribution functions $\rho_i^{(2)}$ and $\rho_i^{(3)}$ are defined as

$$\rho_i^{(2)}(r) = \frac{1}{4\pi}\int \rho_i(r\hat{\mathbf{r}})\, d\hat{\mathbf{r}}, \tag{S11}$$

$$\rho_i^{(3)}(r, s, \theta) = \iint d\hat{\mathbf{r}} d\hat{\mathbf{s}}\; \delta(\hat{\mathbf{r}}\cdot\hat{\mathbf{s}} - \cos\theta)\, \rho_i(r\hat{\mathbf{r}})\, \rho_i(s\hat{\mathbf{s}}), \tag{S12}$$

$$\rho_i(\mathbf{r}) = \sum_{j=1}^{N_\mathrm{a}} f_\mathrm{cut}(|\mathbf{r}_j - \mathbf{r}_i|)\, g(\mathbf{r} - (\mathbf{r}_j - \mathbf{r}_i)) \tag{S13}$$

The function $g$ is the smoothed $\delta$-function, and $f_\mathrm{cut}$ is a cutoff function that smoothly eliminates the contribution from atoms outside a given cutoff radius $R_\mathrm{cut}$. For $\chi_{nl}$ and $P_l$, normalized spherical Bessel functions $\chi_{nl} = j_l(q_n r)$ and Legendre polynomials of order $l$ are used in this work, respectively. For the kernel basis functions, the smooth overlap of atomic positions (SOAP) kernel[22] is employed

$$K(\mathbf{x}_i, \mathbf{x}_{i_\mathrm{B}}) = (\hat{\mathbf{x}}_i \cdot \hat{\mathbf{x}}_{i_\mathrm{B}})^\zeta. \tag{S14}$$

The hat symbol $\hat{\mathbf{x}}_i$ denotes a normalized vector of $\mathbf{x}_i$. The normalization and exponentiation in Eq. (S14) introduce non-linear terms that mix two- and three-body contributions. The parameters of the descriptors and kernel basis functions are listed in Table S1.

One of the critically important advantages of the kernel-based method is that it can provide the necessary information to determine whether the prediction lies within the interpolatable space spanned by the reference points $\{\mathbf{x}_{i_\mathrm{B}} | i_\mathrm{B} = 1, ..., N_\mathrm{B}\}$. This function is essential in TI calculations I and II, where it is necessary to ensure that the trajectories always remain within the interpolatable region to guarantee reversible thermodynamic paths. In this study, we use the spilling factor[21,23] for this determination:

$$s_i = 1 - \frac{\sum_{i_\mathrm{B}}^{N_\mathrm{B}} \sum_{i'_\mathrm{B}}^{N_\mathrm{B}} K(\hat{\mathbf{x}}_i, \hat{\mathbf{x}}_{i_\mathrm{B}})\, K^{-1}(\hat{\mathbf{x}}_{i_\mathrm{B}}, \hat{\mathbf{x}}_{i'_\mathrm{B}})\, K(\hat{\mathbf{x}}_{i'_\mathrm{B}}, \hat{\mathbf{x}}_i)}{K(\hat{\mathbf{x}}_i, \hat{\mathbf{x}}_i)}, \tag{S15}$$

where $K^{-1}$ represents the elements of the inverse of the kernel matrix. If the density of the

reference points is high enough to provide complete overlap among the kernel basis functions, $s_i$ approaches zero, otherwise $s_i$ approaches unity. During MD simulations of various materials,[20,21,24–31] when the structure enters the extrapolated region and catastrophic failures occur, the maximum value of $s_i$ in the unit cell rapidly approaches unity. However, if it remains small (on the order of 0.01, for example), there are no significant errors in the predicted forces or energies, and the resulting structures closely match the FP calculation results. In such cases, the TI calculation yields highly reproducible paths.

The reference points $\{\mathbf{x}_{i_\mathrm{B}}|i_\mathrm{B} = 1, ..., N_\mathrm{B}\}$ and the structures providing training data of energies, forces and stress tensor components are collected on the fly during the active learning MD simulations implemented in VASP.[21,24,32,33] The Bayesian framework allows for accurate predictions of energies, forces, and their uncertainties, enabling efficient on-the-fly sampling of reference structures during MD simulations of target systems. A single MLFF was trained for each surface system explained in Section S5. For each of reactant and product state, a 100 ps annealing simulation from 300 to 500 K and a 100 ps cooling simulation from 500 to 300 K were performed. By the end of the cooling simulation, the MLFF stopped collecting structures and reference points, indicating that it had sufficiently learned the information necessary for the simulations. The numbers of the collected structures ($N_\mathrm{st}$) and the reference points ($N_\mathrm{B}$) for each MLFF are listed in Table S2. Similarly to the previous studies,[20,21,24–31] $N_\mathrm{st}$ is 260-510. As expected, the most complex interface between water and melamine-modified Pt required the most structural configurations. The number of reference points $N_\mathrm{B}$ depends strongly on the element type. As discussed in the previous study,[24] elements like hydrogen, whose surrounding chemical environment varies widely, require numerous reference points. In contrast, elements like copper, situated within a slab where the chemical environment changes little, require fewer reference points. In this way, the on-the-fly active learning algorithm autonomously learns the diversity of the chemical environment for each element during the simulation and automatically determines the necessary number of training data and reference points. Scatter plots of energies and forces predicted by the

MLFFs versus those calculated by the FP method are shown in Fig. S2. In the same figure, the root mean square errors (RMSEs) of the MLFFs for test structures that appear during the TI calculations from the MLFFs to the FP potential energy are also shown. The generated MLFFs achieve root mean square errors of 1.1 to 1.4 meV atom$^{-1}$ for energies and 0.047 to 0.054 eV Å$^{-1}$ for forces, which are comparable to the typical values reported in previous studies.[20,21,24–31] In all FP calculations, exchange-correlation interactions between electrons were modeled using the RPBE+D3 functional.[34–36] The plane-wave cutoff energy was set to 520 eV. For Brillouin zone integration, a 3×3×1 **k**-point mesh was used. The PAW atomic reference configurations were $1s^1$ for H, $2s^22p^2$ for C, $2s^22p^3$ for N, $2s^22p^4$ for O, $3d^{10}4p^1$ for Cu, and $5d^96s^1$ for Pt.[37]

Table S 1: Parameter sets of descriptors and kernel basis functions.

| | | | | | | | | | | | |
|---|---|---|---|---|---|---|---|---|---|---|---|
| $\zeta$ | 4 | $\beta^{(2)}$ | 0.5 | $R_{\mathrm{cut}}^{(2)}$ | 6.0 | $\sigma_{\mathrm{atom}}^{(2)}$ | 0.5 | $N_{\mathrm{R}}^{(2)}$ | 8 | | |
| | | $\beta^{(3)}$ | 0.5 | $R_{\mathrm{cut}}^{(3)}$ | 4.0 | $\sigma_{\mathrm{atom}}^{(3)}$ | 0.5 | $N_{\mathrm{R}}^{(3)}$ | 6 | $L_{\mathrm{max}}^{(3)}$ | 3 |

Table S 2: The number of structures $N_{\mathrm{st}}$ calculated by the FP method to generate training data and the number of kernel basis functions $N_{\mathrm{B}}$ for the MLFFs.

| Material | $N_{\mathrm{st}}$ | $N_{\mathrm{B}}$ (H) | $N_{\mathrm{B}}$ (O) | $N_{\mathrm{B}}$ (Pt) | $N_{\mathrm{B}}$ (Cu) | $N_{\mathrm{B}}$ (C) | $N_{\mathrm{B}}$ (N) |
|---|---|---|---|---|---|---|---|
| Pt | 283 | 6698 | 2630 | 834 | | | |
| Melamine/Pt | 508 | 14403 | 3938 | 1397 | | 1213 | 3845 |
| Pt/$Pt_2Cu$/Pt | 288 | 6830 | 2688 | 71 | 1110 | | |
| Pt/$PtCu_2$/Pt | 259 | 6570 | 2599 | 99 | 1224 | | |
| Pt/Cu/Pt | 282 | 6552 | 2555 | 90 | 1016 | | |

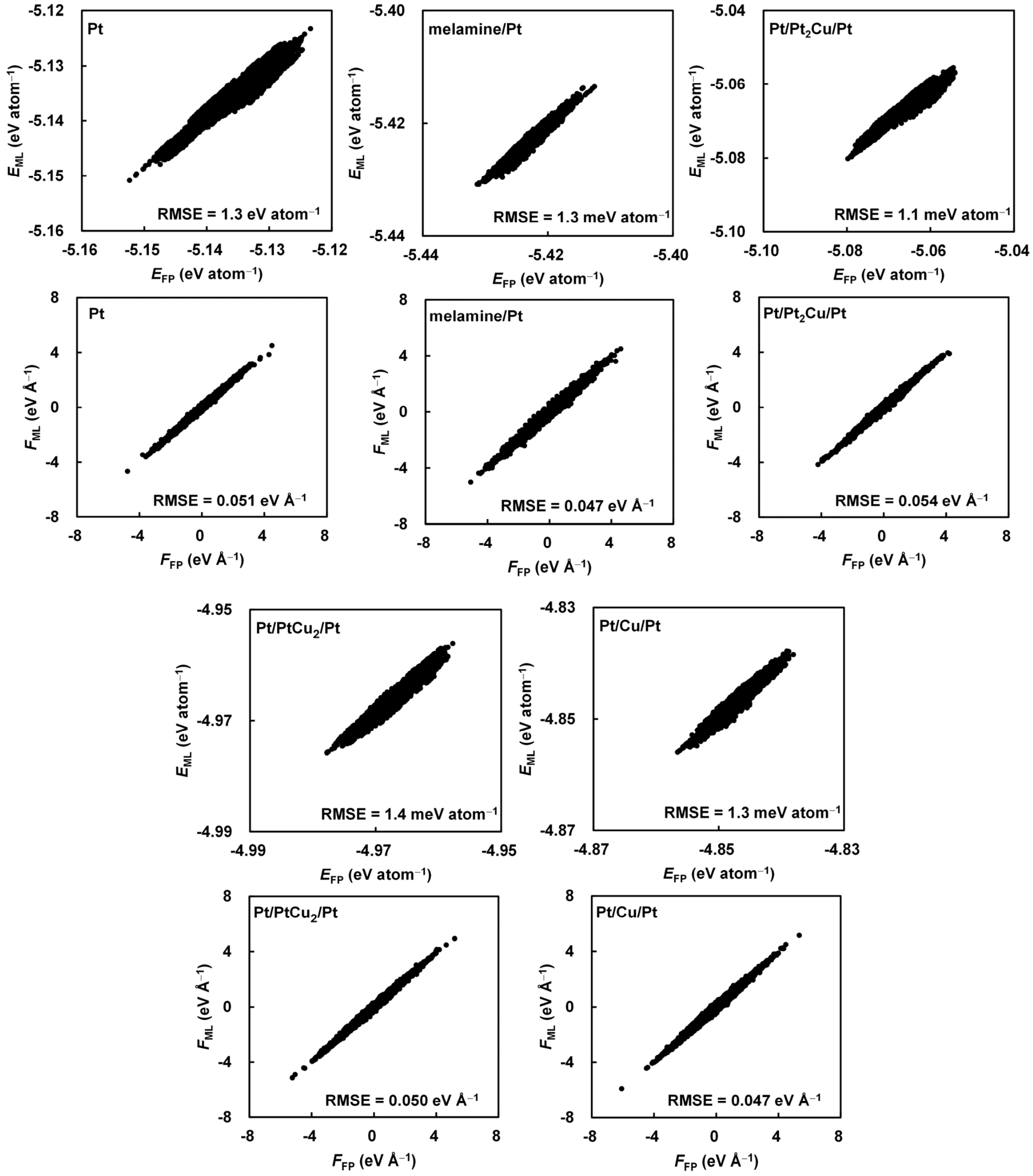


Figure S 2: Scatter plots and RMSEs of energies and forces predicted by the MLFFs compared to the FP results. The accuracy was evaluated on 10,000 structures that appeared during the TI calculations from the MLFF to the FP potential energies.

## S3. Redox potential forming $OH^*$ from $H_2O$

We calculate the redox potentials $U_{\mathrm{OH}}$ on Pt(111) surfaces with and without melamine, and on Pt alloy (111) surfaces. For the calculations, we use the computational standard hydrogen electrode (CSHE) proposed by Nørskov and co-workers.[3,38] This method allows us to calculate the free energies of protons and electrons at electrode potential $U$ versus SHE as

$$A\left[\mathrm{H}^{+}\right]+A\left[\mathrm{e}^{-}\right]=\frac{1}{2}A\left[\mathrm{H}_2\right]-eU, \tag{S16}$$

where $A$ denotes the free energy of the species shown in the square brackets, and $e$ represents the elementary charge. The redox potential is determined by the Gibbs free energy. However, in this study, we approximate it using the Helmholtz free energy obtained from MD simulations in the canonical ensemble,[39,40] as the impact of volume changes is negligibly small. Therefore, the free energy is denoted by $A$ in this context. According to Eq. (S16), the free energy difference of the $OH^*$ formation reaction [backward reaction (5)] can be expressed as follows:

$$\Delta A=A\left[\mathrm{OH}*\right]+\frac{1}{2}A\left[\mathrm{H}_2\right]-eU-A\left[\mathrm{H}_2\mathrm{O}\right]-A\left[*\right]. \tag{S17}$$

At the redox potential $U_{\mathrm{OH}}$, $\Delta A$ is zero. To determine the redox potential, we employ the method introduced by Nørskov, Rossmeisl, and their co-workers.[1,3] This approach assumes that the effects of the interfacial electric field are negligible and approximates the interfacial free energies $A\left[\mathrm{OH}*\right]$ and $A\left[*\right]$ using electrically neutral surfaces, based on a first-order approximation with respect to the surface excess charge.[38] With this approximation, the two interfacial free energies become independent of $U$, reducing Eq. (S17) to a linear function of $U$. As a result, without the need for multiple FP calculations for various charged states to solve the non-linear Eq. (S17), $U_{\mathrm{OH}}$ can be simply calculated using Eq. (6) based solely on

FP calculations for electrically neutral surfaces. This approximation significantly reduces the computational cost, facilitating the application of the MLFF-aided free energy calculation scheme, which requires tens of picoseconds of FPMD simulations to evaluate errors in MLFFs while enabling nanosecond-scale simulations necessary for statistical sampling, as detailed in Section S4. As shown in Section S9, the potentials of zero charge calculated from finite-temperature FPMD simulations indicate that the electric field effect of melamine on the free energy of OH* is minimal. Therefore, the first-order approximation is considered a reasonable approach for assessing the impact of melamine on the stability of OH*.

In the original method,[3] liquid-solid interfaces containing the OH adsorbate (OH*) and surface site (*) were further approximately modelled by ice-like bilayers and single crystal surfaces optimized by FP calculations. Water was represented as an isolated molecule in vapor in equilibrium with liquid water. The free energies of the surface site and OH adsorbate were computed using harmonic oscillator models, and the free energies of hydrogen ($H_2$) and water ($H_2O$) were calculated using ideal gas models at the standard state and empirical saturated vapor pressure at 298 K, respectively. However, as described in the introduction, the effects of solvation structure modified by melamine cannot be evaluated using the ice-like model optimized at 0 K. To solve this problem, we computed the free energy change using a rigorous TI scheme with finite-temperature MD simulations.

# S4 Thermodynamic integration

## S4.1 Overview

As in our previous study,[27,31,41,42] the TI is decomposed into two steps: (1) TI using MLFFs from the non-interacting hydrogen and the liquid-solid interface involving OH* to the fully interacting hydrogen in the interfacial system, and (2) TI from the MLFF potential to the

FP potential:

$$\Delta A_1 = \Delta A^{\mathrm{ML}} + \Delta A^{\mathrm{FP-ML}}. \tag{S18}$$

While the TI step (1) provides a statistically accurate free energy for the MLFF, the MLFF model may introduce a non-negligible error. This error is corrected by the TI step (2). In this step, the potential energy transitions seamlessly from the MLFF to the FP potential. As discussed in the previous studies,[31,41,42] one of the main advantages of our ML-aided scheme is that the initial TI step from the non-interacting to the interacting system is done using the MLFF model. This initial step, which requires the use of an infinitesimally small interaction between the inserted atom and other atoms, becomes extremely challenging as the yet non-interacting atom approaches or even overlaps with other atoms and hence experiences a significant repulsive potential. Additionally, it is highly challenging to perform adequate configurational sampling of the interfacial systems involving slow solvent reorganizations, requiring multiple several ns to tens of ns of simulations along the coupling parameter with the FP method. The MLFF significantly accelerates these computations by several orders of magnitude. Another major advantage is that the accurate reproduction of FP structures by the MLFF results in small and nearly linear integrands with respect to the coupling parameter for the TI step (2), facilitating the convergence of this integral after a few tens of ps of MD simulations, thus reducing the high computational costs associated with FP calculations.

In the modified $\lambda$-MLFF scheme,[27,41,42] TI step (1) is further divided into two steps: (I) from a non-interacting hydrogen atom to a hydrogen atom constrained to a specific OH* by a model potential, and (II) from the model potential to the MLFF:

$$\Delta A^{\mathrm{ML}} = \Delta A_{\mathrm{I}}^{\mathrm{ML}} + \Delta A_{\mathrm{II}}^{\mathrm{ML}}, \tag{S19}$$

$$\Delta A_{\mu}^{\mathrm{ML}} = \int_0^1 \left\langle \frac{\partial H_{\mu}^{\mathrm{ML}}}{\partial \lambda_{\mu}} \right\rangle_{\lambda_{\mu}} d\lambda_{\mu} \ (\mu = \mathrm{I} \ \mathrm{or} \ \mathrm{II}) \, . \tag{S20}$$

Details of the Hamiltonian $H_{\mu}^{\mathrm{ML}}$ and the model potential are explained in subsection S4.2. The key point of this scheme is to avoid erroneous predictions by the MLFF on physically meaningless structures, which could lead to the irreversible trapping of the system in false virtual wells during the TI simulation.

The TI step (2) is conducted on both the reactant state ($\kappa = 0$) and the product state ($\kappa = 1$) along the coupling constant $\eta$ that seemlessly connects the MLFF and the FP potential energy surfaces:

$$\Delta A^{\mathrm{FP-ML}} = \Delta A_{1}^{\mathrm{FP-ML}} - \Delta A_{0}^{\mathrm{FP-ML}}, \tag{S21}$$

$$\Delta A_{\kappa}^{\mathrm{FP-ML}} = \int_{0}^{1} \left\langle \frac{\partial H_{\kappa}^{\mathrm{FP-ML}}}{\partial \eta} \right\rangle_{\eta} d\eta. \tag{S22}$$

Details of the Hamiltonian $H_{\kappa}^{\mathrm{FP-ML}}$ is also explained in subsection 4.2.

Finally, the influence of nuclear quantum effects on the free energy change was estimated using the difference between the quantum oscillator model and the classical oscillator model.[41,42] Details of the nuclear quantum effects are provided in subsection S4.3.

In addition to the TI calculations of the free energy difference $\Delta A_{\mathrm{OH}}$ at the liquid-solid interface, we also evaluated $\Delta A_{\mathrm{OH}}$ on surfaces in vacuum using conventional structural optimization and the harmonic oscillator model[3] for comparison. Details are provided in Section S7.

## S4.2. Details of TI

The Hamiltonian $H_{\mu}^{\mathrm{ML}}$ in Eq. (S20) is represented as:

$$H_{\mathrm{I}}^{\mathrm{ML}} = \sum_{i=1}^{N_{\mathrm{a}}} \frac{|\mathbf{p}_i|^2}{2m_i} + \lambda_{\mathrm{I}} U_{\mathrm{model}} + \sum_{i \notin \mathrm{M}} U_i\left(0\right), \tag{S23}$$

$$H_{\mathrm{II}}^{\mathrm{ML}} = \sum_{i=1}^{N_{\mathrm{a}}} \frac{|\mathbf{p}_i|^2}{2m_i} + \lambda_{\mathrm{II}} \sum_{i=1}^{N_{\mathrm{a}}} U_i(1) + (1-\lambda_{\mathrm{II}}) \left[ U_{\mathrm{model}} + \sum_{i \notin \mathrm{M}} U_i\left(0\right) \right], \tag{S24}$$

where $N_{\mathrm{a}}$ is the number of atoms, $\mathbf{p}_i$ is the momentum vector of $i$-th atom, and the symbol M denotes the inserted species, which in this study are two hydrogen atoms. As explained in Section S2 regarding the kernel-based approach, the atomic potential energies $[U_i(0)$ and $U_i(1)]$ are expressed as a linear combination of functions $K(\mathbf{x}_i(\lambda), \mathbf{x}_{i_{\mathrm{B}}})$, similar to what was described earlier:

$$U_i(\lambda) = \sum_{i_{\mathrm{B}}=1}^{N_{\mathrm{B}}} w_{i_{\mathrm{B}}} K(\mathbf{x}_i(\lambda), \mathbf{x}_{i_{\mathrm{B}}}). \tag{S25}$$

Here, the descriptors $\mathbf{x}_i(\lambda) = \mathbf{x}_i[\rho_i(\mathbf{r}, \lambda)]$ are the functional of the density distribution function around atom $i$,

$$\begin{aligned}\rho_i(\mathbf{r}, \lambda) = &\sum_{j \notin \mathrm{M}} f_{\mathrm{cut}}(|\mathbf{r}_j - \mathbf{r}_i|)\, g(\mathbf{r} - (\mathbf{r}_j - \mathbf{r}_i)) \\ &+ \lambda \sum_{j \in \mathrm{M}} f_{\mathrm{cut}}(|\mathbf{r}_j - \mathbf{r}_i|)\, g(\mathbf{r} - (\mathbf{r}_j - \mathbf{r}_i)).\end{aligned} \tag{S26}$$

Hence, $U_i(0)$ and $U_i(1)$ give the local energies without and with the hydrogen atoms. $U_{\mathrm{model}}$ is the model potential that constrains each hydrogen atom between a specific OH* and water molecule as illustrated in Fig. S3. Its form is represented as

$$U_{\mathrm{model}} = \sum_{i \in \mathrm{M}} \sum_{j \notin \mathrm{M}} a_0 e^{-\left(\frac{r_{ij}}{r_0}\right)^n} + \sum_{i \in \mathrm{M}} \sum_{j \in \mathrm{W}} a_j \left[1 - e^{-b_j(r_{ij} - r_j)}\right], \tag{S27}$$

where the summation over $j$ in the first term is taken over all atoms except for the hydrogen atoms, while the summation over $j$ in the second term is taken over the oxygen atoms in the OH* and water denoted as the symbol W. The first term provides the repulsive potential between each hydrogen atom and other atoms, and the second term provides the Morse potentials between each hydrogen atom and the oxygen atoms in the OH* and water. As in previous studies,[27,31,41,42] the parameters of the model potential were set to the values shown in Fig. S3 to roughly reproduce the water dimer structures obtained by the MLFF.

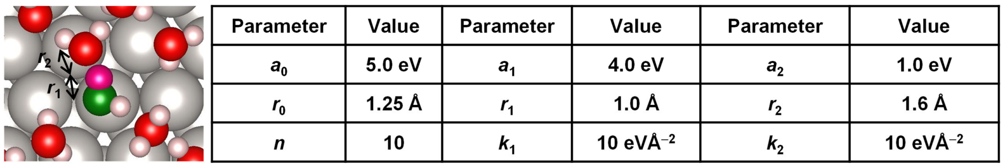


| Parameter | Value | Parameter | Value | Parameter | Value |
|---|---|---|---|---|---|
| $a_0$ | 5.0 eV | $a_1$ | 4.0 eV | $a_2$ | 1.0 eV |
| $r_0$ | 1.25 Å | $r_1$ | 1.0 Å | $r_2$ | 1.6 Å |
| $n$ | 10 | $k_1$ | 10 eVÅ$^{-2}$ | $k_2$ | 10 eVÅ$^{-2}$ |

Figure S 3: Parameters of the model potential. Here, the constrained hydrogen atom is represented as a pink sphere, and oxygen atom in OH* is represented as a green sphere. The parameter $b_j$ is calculated as $\sqrt{k_j/(2a_j)}$.

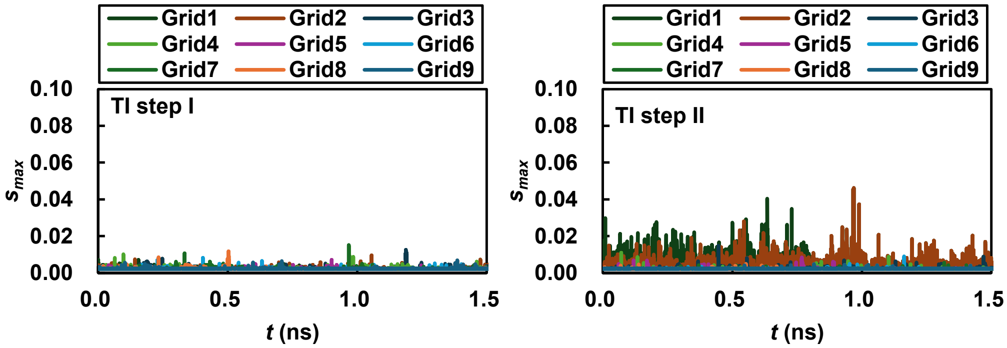


Figure S 4: The maximum spilling factor in the unit cell as a function of the MD simulation time for each grid (1 through 9) during the TI steps I and II for the melamine/Pt system, which is the most complex system in this study and required a large amount of training data.

At $\lambda_{\mathrm{I}} = 0$, the Hamiltonian $H_{\mathrm{I}}^{\mathrm{ML}}$ generates the machine-learned potential for the non-interacting hydrogen atoms and the remaining interacting system. As the coupling constant $\lambda_{\mathrm{I}}$ increases, the model potential gradually comes into effect. During this transient process, unrealistic structures may appear near the non-interacting limit ($\lambda_{\mathrm{I}} = 0$). However, since the MLFF $\sum_{i \notin \mathrm{M}} U_i(0)$ ignores the inserted hydrogen atoms, it can accurately predict the potential energy of the remaining interacting system, as can be seen from the snapshot in Fig. 2. The Hamiltonian $H_{\mathrm{II}}^{\mathrm{ML}}$ gradually replaces the model potential with the MLFF $\sum_{i=1}^{N_{\mathrm{a}}} U_i(1)$ for the fully interacting hydrogens. Because the model potential roughly represents the surrounding structure of the fully interacting hydrogens, the MLFF can accurately predict the potential energy of the entire system. As shown in Fig. S4, the spilling factor remains small throughout the trajectories of TI steps I and II, indicating that all predictions fall within the interpolation region of the training data.

Similar to previous studies, the variable transformations for the coupling constants $\lambda_{\mathrm{I}}$ and $\lambda_{\mathrm{II}}$ in the integrations in Eq. (S20) were performed as:

$$\lambda_{\mathrm{I}} = \left(\frac{x_{\mathrm{I}}+1}{2}\right)^{\frac{1}{1-k}}, \tag{S28}$$

$$\lambda_{\mathrm{II}} = 1 - \left(\frac{x_{\mathrm{II}}+1}{2}\right)^{\frac{1}{1-k}}, \tag{S29}$$

where $k$ was set to 0.7. Numerical integrations were conducted using the 10-point Gauss-Lobatto quadrature method for the variables $x_{\mathrm{I}}$ and $x_{\mathrm{II}}$.[25,27] In this setting, MD simulations are required at 9 grid points since the weight at one endpoint of the grids is zero. At each grid point, 80 ps MD simulations were conducted 19 times, with initial structures exchanged between adjacent grids. The total simulation time across all grids amounted to 27.4 ns. This simulation resulted in sufficiently small statistical errors to evaluate the effects of melamine and alloying.

The Hamiltonian $H_{\kappa}^{\mathrm{FP-ML}}$ in Eq. (S22), used to calculate the free energy difference between the MLFF and FP potential energies, is defined as

$$H_{\kappa}^{\mathrm{FP-ML}} = \sum_{i=1}^{N_{\mathrm{a}}} \frac{|\mathbf{p}_i|^2}{2m_i} + \eta U_{\kappa}^{\mathrm{FP}} + (1-\eta)\, U_{\kappa}^{\mathrm{ML}}, \tag{S30}$$

where $U_{\kappa}^{\mathrm{FP}}$ and $U_{\kappa}^{\mathrm{ML}}$ are the potential energies calculated by the FP method and the MLFFs. Numerical integrations along the coupling constant $\eta$ were performed using the Simpson rule with five equally spaced grid points. Thanks to the accurate MLFFs, even a short 10 ps MD simulation at each grid point achieved small statistical error bars.

## S4.3. Nuclear quantum effects and ideal gas model

Similarly to the previous studies,[43,44] the influence of nuclear quantum effects on the free energy change was estimated using the difference between the quantum oscillator model and

the classical oscillator model.

$$A_{\mathrm{q-c}} = A_{\mathrm{q,vib}} - A_{\mathrm{c,vib}}, \tag{S31}$$

$$A_{\mathrm{q,vib}} = \sum_i \frac{h\nu_i}{2} + \sum_i k_{\mathrm{B}}T\ln\left(1 - e^{-\frac{h\nu_i}{k_{\mathrm{B}}T}}\right), \tag{S32}$$

$$A_{\mathrm{c,vib}} = \sum_i k_{\mathrm{B}}T\ln\left(\frac{h\nu_i}{k_{\mathrm{B}}T}\right), \tag{S33}$$

where $h$ is the Planck constant, and $\nu_i$ is the vibrational frequency of the $i$-th normal mode. The difference in $\Delta A_{\mathrm{q-c}}$ between the reactant state OH* and the product state $H_2O$ was calculated using the vibrational frequencies $\nu_i$ of OH* and $H_2O$ at the interface between the ice-like water bilayer and the Pt(111) surface, as shown in Fig. S5. In these calculations, the interfacial models were optimized, and Γ-phonons were calculated by diagonalizing the partial Hessian matrices of OH* and $H_2O$, as indicated by the dotted circles in Fig. S5. The final nuclear quantum effect on $\Delta A_1$ was calculated as $\Delta A_{\mathrm{q-c}} = A_{\mathrm{q-c}}\,[\mathrm{H_2O}] - A_{\mathrm{q-c}}\,[\mathrm{OH*}] = 0.240$ eV, where $A_{\mathrm{q-c}}\,[.]$ denotes the $A_{\mathrm{q-c}}$ of the species indicated in the square brackets. The vibrational frequencies and free energies are presented in Table S3.

The difference in free energy due to the volume difference between the unit cell used in the simulations and the same hydrogen gas at standard state was determined using the following ideal gas free energy difference:

$$A_{\mathrm{corr}} = k_{\mathrm{B}}T\ln\left[\frac{V_0^N}{\Lambda^{3N}N!}\right] - k_{\mathrm{B}}T\ln\left[\frac{V_{\mathrm{cell}}^N}{\Lambda^{3N}N!}\right], \tag{S34}$$

Here, $N$ is the number of gaseous hydrogen atoms, $V_0$ is the volume (24.45 L mol$^{-1}$) at the standard condition, $\Lambda$ is the thermal de Broglie wavelength, and $V_{\mathrm{cell}}$ is the volume of the unit cell used in the computations.

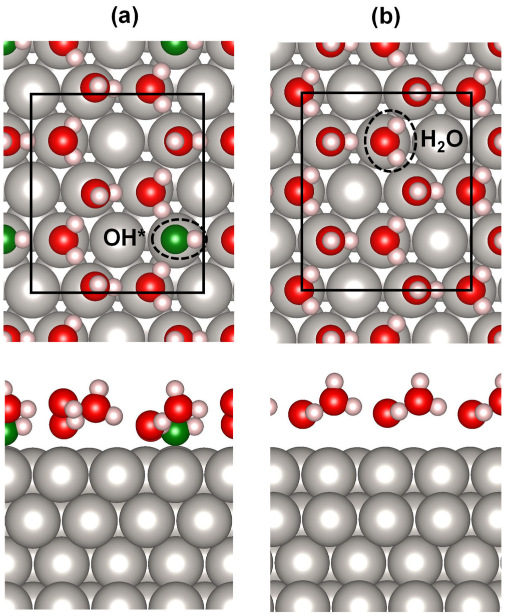


Figure S 5: Interfacial models used to compute the vibrational frequencies $\nu_i$.

Table S 3: Nuclear quantum effects on the free energies of OH∗ and $H_2O$ at the interface between the water bilayer and Pt(111) surface. The units of free energy and vibrational frequencies are eV and $cm^{-1}$, respectively.

| Property | OH* | $H_2O$ |
|---|---|---|
| $\nu_i$ | 3571 | 3594 |
| | 958 | 3503 |
| | 785 | 1621 |
| | 389 | 640 |
| | 258 | 556 |
| | | 541 |
| | | 199 |
| | | 152 |
| | | 53 |
| $F_{\mathrm{q,vib}}$ | 0.374 | 0.642 |
| $F_{\mathrm{c,vib}}$ | 0.073 | 0.102 |
| $F_{\mathrm{q-c}}$ | 0.300 | 0.540 |

## S5 Interfacial models

The interfaces between water and pure Pt(111) surfaces, with and without melamine, and monolayer Pt skins on Pt alloy (111) surfaces are modeled as shown in Fig. S6. As explained in Sections S1 and S3, this study adopts the method introduced by Nørskov, Rossmeisl, and their co-workers[1,3] for calculating the redox potential of OH formation and its application to predicting ORR activity. This approach assumes that the effect of interfacial electric fields is small and calculates redox potentials using electrically neutral surface models, based on a first-order approximation with respect to the surface excess charge.[38] Accordingly, the solid surfaces were approximately modeled using electrically neutral 4-layer slabs with a $3{\times}2\sqrt{3}$ periodicity. For Pt alloys, we evaluated the near-surface alloys Pt/$Pt_{1-x}Cu_x$/Pt(111) ($x$=1/3, 2/3, and 1). In a previous experimental and theoretical study,[15] these near-surface alloy single crystals with well-controlled structure and composition were synthesized by annealing Pt(111) surfaces with Cu adsorbates deposited by underpotential deposition, and the redox potentials forming OH* and the ORR rate on these surfaces were experimentally identified. Additionally, FP calculations using the conventional harmonic oscillator model and structures optimized at 0 K were also performed on these surfaces, providing good comparison points for our results. Furthermore, these surfaces adopt a low-spin state, facilitating the convergence of FP calculations. Angular resolved X-ray photoemission spectroscopy conducted in the previous study revealed that Cu atoms are primarily located in the second layer from the surface. The amount of Cu in the second layer was controlled at 1/4 to 1 monolayer by varying the amount of deposited Cu atoms, while the other layers were mainly composed of Pt atoms. The models in Fig. S6 (c) to (e) represent the observed Cu distribution in the second layer while the third layer of the symmetrical slab models used in the calculations does not fully represent the elemental distribution of the inner pure Pt layers in the experimental samples.

One OH adsorbate per unit cell was located on each side of the slab, resulting in an OH coverage of 1/12 ML, and two hydrogen atoms were inserted to generate two additional water

molecules at the interfaces. By using a symmetrical arrangement and calculating the two H insertion reactions, the free energy change per cell due to the reaction can be increased, thereby reducing numerical noise in the desired properties.

To represent the melamine-modified surface, one melamine molecule per unit cell was directly adsorbed on each side of the Pt(111) surface. As a result, the surface coverage of melamine molecules was 1/12 ML. This coverage falls within the experimental range of 0.04 to 0.12 ML, depending on surface defects and the concentration of melamine in the solution.[16]

As detailed in Section S8 of SI, preliminary calculations using finite-temperature MD simulations with MLFF and conventional FP calculations using harmonic oscillator models indicated that, under the cathode potential of PEMFCs and in water, melamine adsorbs differently compared to the structure found on Pt(111) in a vacuum.[45] As shown in Fig. S6 (b), the six-membered ring composed of C and N is nearly perpendicular to the Pt surface, with melamine adsorbed via two amino groups. Furthermore, these preliminary calculations indicated that melamine stabilizes in an oxidized state with one hydrogen removed from each of the two amino groups bound to Pt. Based on these results, we modeled the melamine-modified Pt(111) surface as shown in Fig. S6 (b).

The interfacial model was constructed through the following process. The lattice parameter of the Pt and Pt alloy slabs was set to 4.03 Å , based on the bulk pure Pt calculated by the FP method. Melamine molecules were placed on both sides of the Pt(111) slab. Initially, the positions of the adsorbed melamine molecules were set according to the optimized structure reported in the previous study.[45] However, as explained in Section S8, it was found that the structure shown in Fig. S10 (e) is more stable under high potential (0.6-1.0 V) ORR conditions in water, so this structure was used in the calculations. A water layer was inserted into the vacuum gap between the slabs. To prepare the initial water configuration, a 1 ns classical MD simulation was conducted on the bulk liquid water using the polymer consistent force field (PCFF).[46] The equilibrated water layer was then inserted

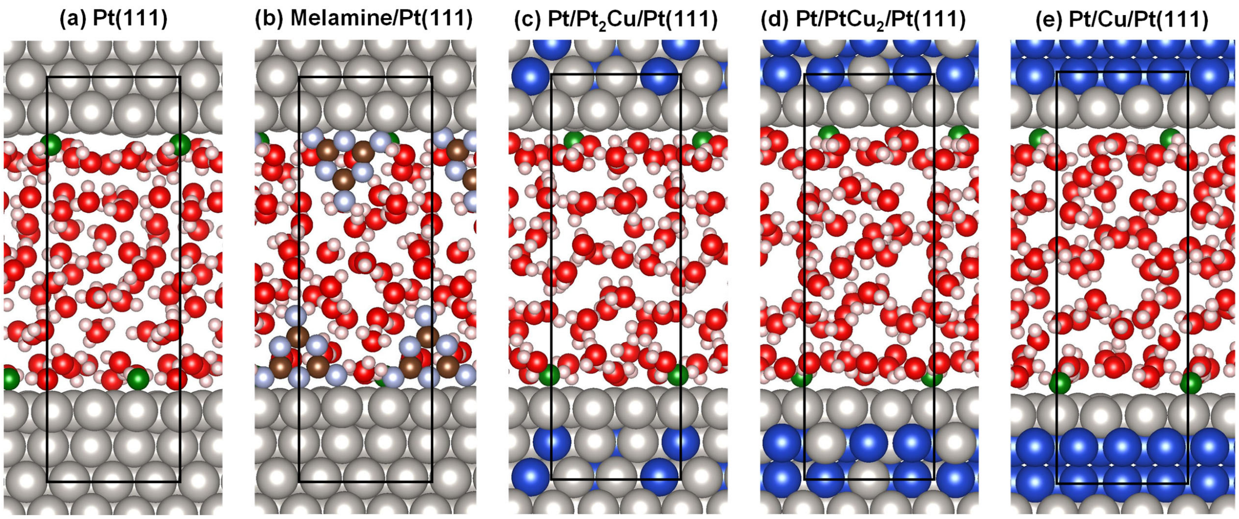


Figure S 6: Models of interfaces between water and (a) Pt(111), (b) Pt(111) with melamine, (c) Pt/$Pt_2Cu$/Pt(111), (d) Pt/$PtCu_2$/Pt(111), and (e) Pt/Cu/Pt(111) surfaces. Small white spheres represent H atoms, brown medium spheres represent carbon atoms, light blue medium spheres represent nitrogen atoms, red medium spheres represent oxygen atoms in $H_2O$, green medium spheres represent oxygen atoms in OH adsorbates, blue large spheres represent Cu atoms, and silver large spheres represent Pt atoms. The squares show the unit cells.

between the slabs. For the Pt(111) surface with melamine, water molecules overlapping with the melamine molecules were removed. The number of water molecules was set to 48 for the Pt and Pt alloy slabs without melamine, and 38 for the Pt slabs with melamine. After further relaxing the interfacial structures with 100 steps of structural optimization using the FP method, active-learning MD simulations were performed to generate the MLFFs. After generating the MLFFs, the length of the unit cell along the direction perpendicular to the surface was equilibrated using a 100 ps $NP_zT$ ensemble MD simulation with the generated MLFFs. The cell length was then adjusted to the equilibrated value, and production runs were performed using these structures as the initial configurations.

# S6. PDOS, d-band centers and Bader charges

Figure S7 shows the PDOS, d-band centers, and Bader charges of surface Pt atoms (sites 1 to 3) for the melamine/Pt system and Pt without melamine. Among these, only the Pt atom (site 3) located beneath the melamine molecule exhibits noticeable changes in these properties compared to the Pt atoms on the bare Pt surface.

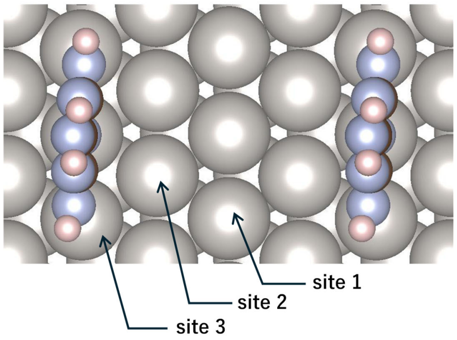


| | $\varepsilon_d$ | $Q$ |
|---|---|---|
| Pt | −1.912 | −0.046 |
| Melamine/Pt (site 1) | −1.920 | −0.036 |
| Melamine/Pt (site 2) | −1.970 | −0.029 |
| Melamine/Pt (site 3) | −2.385 | 0.228 |
| Melamine/Pt (melamine) | | −0.690 |

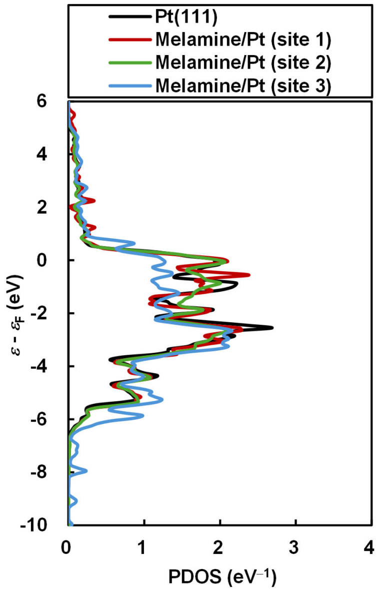


Figure S 7: Projected density of states (PDOS) for the d-orbitals, d-band centers [$\epsilon_d$ (eV)], and Bader charges [$Q$ (e)] of surface Pt atoms (sites 1 to 3) in the melamine/Pt system and Pt without melamine. In the finite-temperature MD simulation in water, OH is adsorbed at site 1.

## S7. Free energy difference on surface in vacuum

To investigate solvation effects, the free energy differences ($\Delta A_{\mathrm{OH}}$) on five surfaces in vacuum were also evaluated. Since the free energies of isolated molecules and adsorbates in a vacuum are accurately represented by the harmonic oscillator model and ideal gas model, the evaluation was conducted based on the conventional method of structural optimization and frequency analysis using the FP method.[3] The interfacial free energies $A\,[\mathrm{OH}*]$ and $A\,[*]$ in Eq. (S17) were calculated using the harmonic oscillator model, while the free energies of the molecules $A\,[\mathrm{H_2}]$ and $A\,[\mathrm{H_2O}]$ were calculated using the ideal gas model. The five surfaces in vacuum were modeled by slabs without water molecules, as shown in Fig. S8. Similar to the slab models used for the TI calculations at the solid-liquid interface, one OH adsorbate was placed on each side of the slab. The initial positions of the OH adsorbates were set to those in the final structure obtained at the non-interacting limit ($\lambda_{\mathrm{I}} = 0$) in TI step I for the solid-liquid interface, where the OH adsorbates were stably formed. The surfaces with and without the OH adsorbate were optimized with a force threshold of 0.02 eV Å$^{-1}$, and their Γ-phonons were calculated by diagonalizing the partial Hessian matrices of the OH adsorbate. Similarly, $H_2$ and $H_2O$ molecules placed in a cubic unit cell with a side length of 15 Å were also optimized, and their Γ-phonons were calculated. The temperature was set to 298 K, with the gaseous pressure of $H_2$ set to 0.1 MPa and that of $H_2O$ set to the empirical vapor pressure of 0.0035 MPa, consistent with the previous study.[3] The parameters for the FP calculations were the same as those described in Section S2.

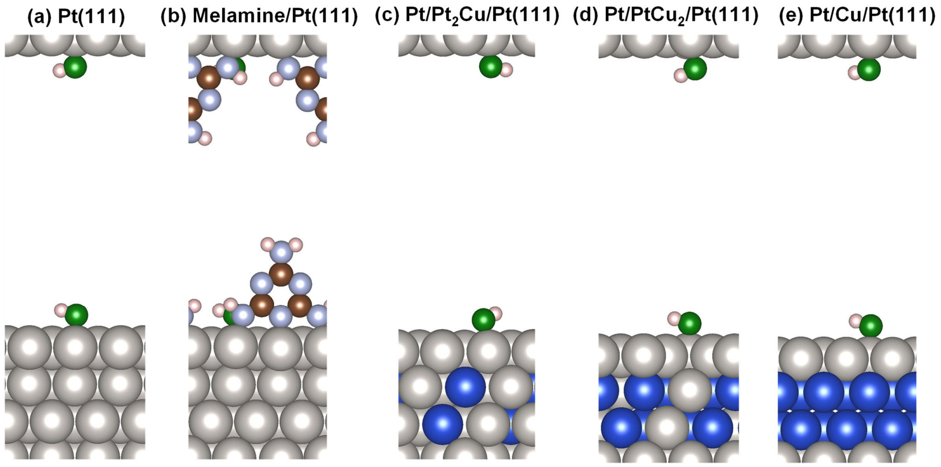


Figure S 8: Unit cells of the slab models used to compute the free energy differences $\Delta A_{\mathrm{OH}}$ on the five surfaces in vacuum.

## S8. Adsorption structure of melamine

We started the MD simulations using the adsorption structure of melamine on the Pt(111) surface in a vacuum proposed by Tada and co-workers,[45] who explored 10,648 structures via FP calculations. In the proposed structure, melamine is bound to the Pt surface through one nitrogen atom in the triazine ring and one nitrogen atom in the amino ($NH_2$) group, making the triazine plane parallel to the Pt surface [see the structure at $t$=0 ps in Fig. S9 or Fig. S10 (a)]. However, our MD simulations of the adsorbates in water indicated that the structure identified in a vacuum is unstable under ORR conditions, as explained below.

In our simulation, we placed 38 water molecules on top of the adsorbed melamine molecules and relaxed the structure by 100 step structural optimizations using the FP calculation. From the relaxed structure, we conducted the 200 ps heating and annealing MD simulations to generate the MLFF on the fly. Subsequently, we further trained the MLFF on the interfacial system containing OH adsorbates, which were generated by removing hydrogen atoms, via the 200 ps heating and annealing MD simulations. During this latter heating simulation, we observed that one hydrogen atom in the amine ($NH_2$) transfers to the OH adsorbate to form water molecule. As shown in Fig. S9, after the hydrogen transfer, the adsorbed melamine standed upright, with its trazine plane perpendicular to the solid surface. We conducted two additional simulations starting from different initial positions of OH adsorbates and observed very similar structural changes. The result likely indicates that the amine group of the adsorbed melamine loses one hydrogen atom to form the partially oxidized state at the electrode potential of ORR, where OH adsorbates are formed on the Pt surface.

To verify this observation, we evaluated the free energies of the adsorbed melamine molecule in several oxidation states as a function of the electrode potential. The reaction free energy to form a particular oxidized state of the adsorbed melamine molecule was

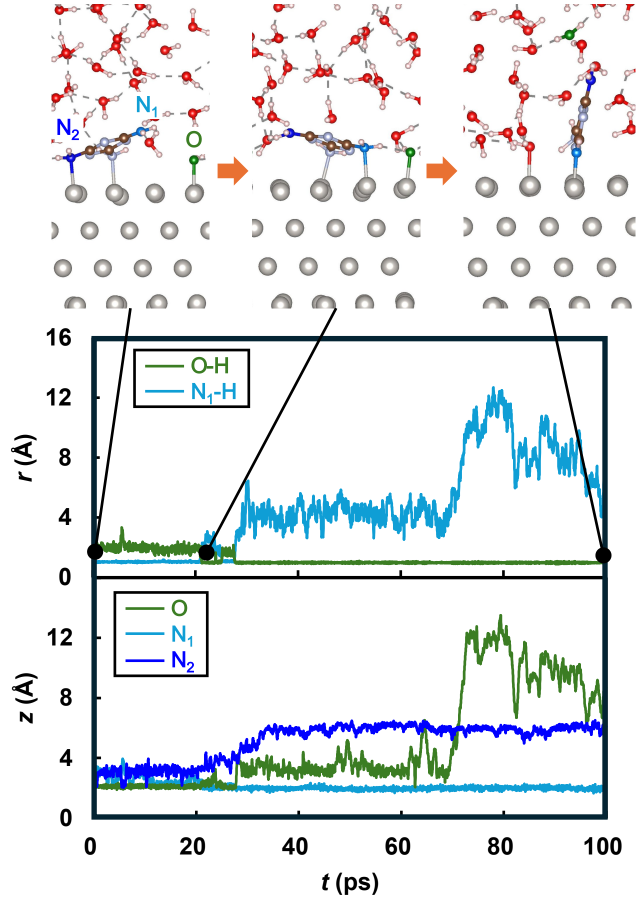


Figure S 9: Hydrogen transfer from the $NH_2$ group to the OH adsorbate to form the water molecule and the change in the adsorption structure of the melamine observed during the 100 ps heating MD simulation.

calculated similarly to Eq. (S17), as

$$\Delta A = A\left[\mathrm{R-NH}_{2-y}\right] + \frac{y}{2}A\left[\mathrm{H}_2\right] - yeU - A\left[\mathrm{R-NH}_2\right], \tag{S35}$$

where $\mathrm{R-NH}_{2-y}$ denotes the melamine with the $\mathrm{NH}_{2-y}$ group.

Here, for this preliminary examination, instead of the rigorous but computationally expensive TI scheme explained in Section S4, we calculated the free energies of adsorbed melamine molecules in vacuum using the harmonic oscillator model.[3] In this calculation, we determined the adsorption structures of melamine in various oxidation states in a vacuum by structural optimization using the FP method. Additionally, we calculated the Γ-phonon

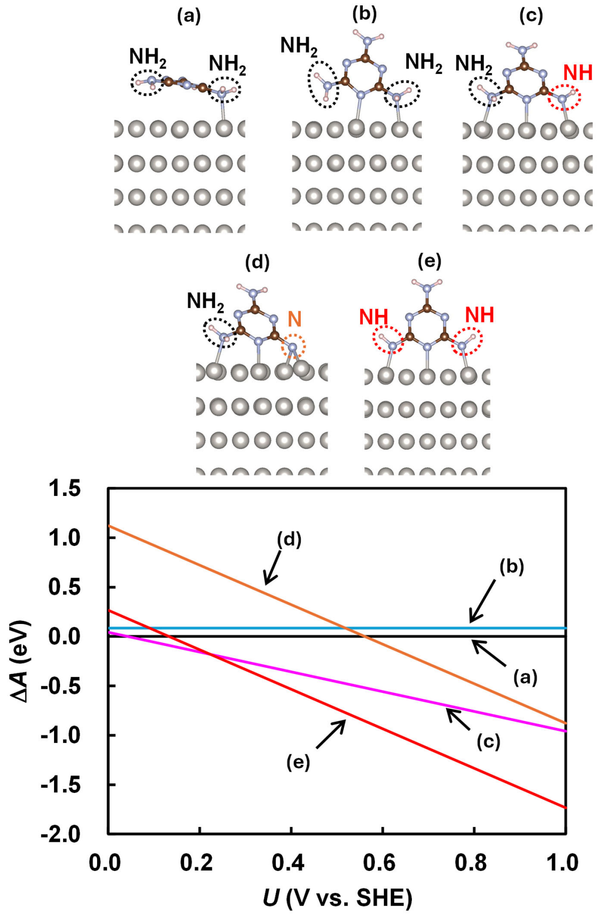


Figure S 10: Adsorption structures of the melamine molecule at several oxidation states, and their free energies as functions of the electrode potential $U$.

of these structures using FP calculations to determine the free energies of the melamine molecules. The results are shown in Fig. S10. The reference for the free energy is set to the melamine adsorbed with two unoxidized $NH_2$ groups, as shown in Fig. S10 (a), which was suggested to be stable in vacuum under ambient conditions.[45] The vertical axis, $\Delta A$, indicates the change in free energy from this reference state when forming the specified state at potential $U$. As reported by Tada et al.,[45] comparing the results for the non-oxidized melamine with two $NH_2$ groups in adsorption structures (a) and (b) shows that the adsorption structure (a), where the triazine plane is parallel to the Pt surface, has lower free energy than structure (b), where the plane is perpendicular to the surface, in vacuum. However, at

potentials higher than 0.2 V vs. SHE, the oxidized melamine with two NH groups has lower free energy. Additionally, in this oxidized state, unlike the structure (a) of non-oxidized melamine in a vacuum, the triazine ring's plane is oriented perpendicular to the Pt surface, as observed in the MD simulations. From these results, we conclude that under high potential (0.6-1.0 V) ORR conditions, the $NH_2$ groups of melamine are oxidized to form NH groups, adopting an adsorption structure where the molecular plane is perpendicular to the surface.

## S9. Potential of zero charge

To verify the applicability of the first-order approximation proposed by Nørskov and co-workers[3,38] (see Section S3), we evaluated the effect of adsorbed melamine on the potential of zero charge (PZC) of the Pt surface in water using the alignment method proposed in previous studies.[31,41,47] The details are explained below.

In electrochemistry, the potential of the vacuum level just outside the water surface is used as the common reference point. However, in codes employing periodic boundary conditions, it is standard practice to set the average electrostatic potential to zero. Consequently, all calculated eigenvalues are implicitly referenced to this zero-potential point. The potential difference $\Delta\phi$ between the vacuum level and the zero-potential point of the periodic system can be determined through a separate calculation involving the interface between water and vacuum. In practical calculations, as schematically shown in Fig. S11, the $\Delta\phi$ between the water-vacuum interface and the water-Pt interface is corrected using the O 1s levels as a common reference point for both systems. By this alignment, $\Delta\phi$ is calculated as:

$$e\Delta\phi = \mu - \epsilon_{1\mathrm{s,w/v}} + \epsilon_{1\mathrm{s,w/s}}, \tag{S36}$$

where $\mu$ represents the vacuum level, $\epsilon_{1\mathrm{s,w/v}}$ denotes the O 1s level of water molecules at the center of the water slab in vacuum, and $\epsilon_{1\mathrm{s,w/s}}$ refers to the O 1s level of water molecules at the center of the water layer far from the Pt surface (for details of the derivation, see Refs.[31,41]). The PZC scaled to the vacuum level is given by $-(\epsilon_\mathrm{F} - e\Delta\phi)/e$, where $\epsilon_\mathrm{F}$ represents the Fermi level of the water-Pt interface. Using this method, the PZCs of Pt(111) surfaces covered with liquid water were calculated both with and without the presence of melamine. For each system, 200 configurations were randomly selected from the 10-ps FPMD trajectory, and the averages of the Fermi energy and O 1s levels were computed. The O 1s levels were calculated for water molecules located within ±1 Å of the center of the water layer.

The calculations yield PZCs of 4.78±0.03 V and 5.01±0.02 V on the vacuum scale for

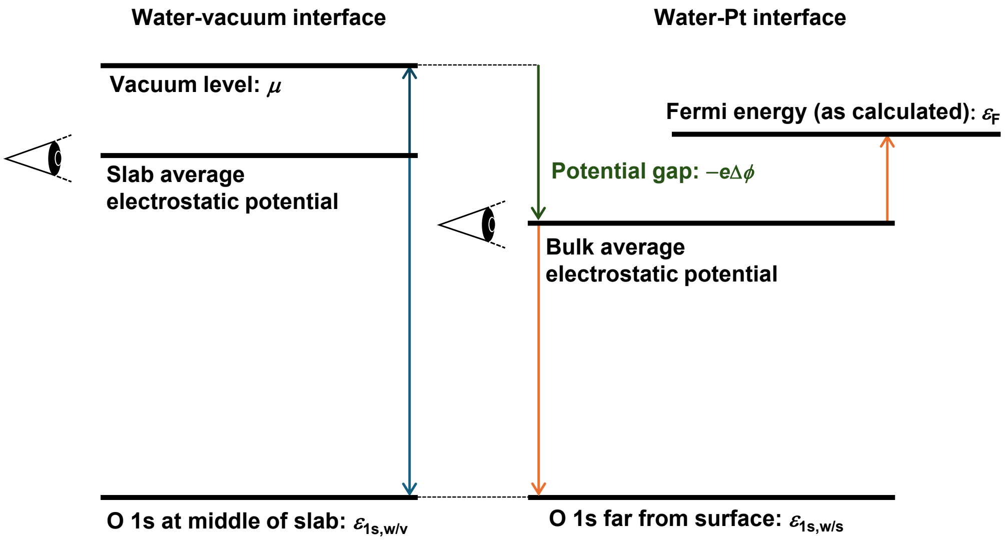


Figure S 11: Electron energy alignment for calculating the potential of zero charge of metal surfaces covered with liquid water.

Pt(111) surfaces without and with melamine, respectively. Using the experimental absolute standard hydrogen electrode potential (ASHEP) of 4.44±0.02 V,[48] these values are converted to 0.34±0.04 V and 0.57±0.03 V versus SHE for the Pt(111) surfaces without and with melamine, respectively. The former value for the pristine Pt(111) surface in water is in good agreement with the experimental value of 0.23±0.08 V versus SHE.[49]

Accordingly, the change in the interfacial electric field at a constant electrode potential caused by melamine can be estimated as $(0.57\ \text{V} - 0.34\ \text{V})/3\ \text{Å} = 0.08\ \text{V Å}^{-1}$, assuming that the width of the double layer is approximately 3 Å as in Ref.[3] Using the dipole moment of 0.05 e Å for OH* reported in Ref.,[3] the effect of this electric field change on the free energy of OH* is estimated to be $0.05\ \text{e Å} \times 0.08\ \text{V Å}^{-1} = 4$ meV. This estimation suggests that the first-order approximation, which has been successfully applied to a wide range of electrocatalytic reactions,[2,50–58] is also a reasonable approach for evaluating the effect of melamine on the free energy of OH*.

# References


(1) Rossmeisl, J.; Karlberg, G. S.; Jaramillo, T.; Nørskov, J. K. Steady state oxygen reduction and cyclic voltammetry. *Faraday Discuss.* **2009**, *140*, 337–346.

(2) Kulkarni, A.; Siahrostami, S.; Patel, A.; Nørskov, J. K. Understanding Catalytic Activity Trends in the Oxygen Reduction Reaction. *Chemical Reviews* **2018**, *118*, 2302–2312.

(3) Nørskov, J. K.; Rossmeisl, J.; Logadottir, A.; Lindqvist, L.; Kitchin, J. R.; Bligaard, T.; Jónsson, H. Origin of the Overpotential for Oxygen Reduction at a Fuel-Cell Cathode. *The Journal of Physical Chemistry B* **2004**, *108*, 17886–17892.

(4) Jinnouchi, R.; Kodama, K.; Hatanaka, T.; Morimoto, Y. First principles based mean field model for oxygen reduction reaction. *Phys. Chem. Chem. Phys.* **2011**, *13*, 21070–21083.

(5) Hansen, H. A.; Viswanathan, V.; Nørskov, J. K. Unifying Kinetic and Thermodynamic Analysis of 2 e– and 4 e– Reduction of Oxygen on Metal Surfaces. *The Journal of Physical Chemistry C* **2014**, *118*, 6706–6718.

(6) Abild-Pedersen, F.; Greeley, J.; Studt, F.; Rossmeisl, J.; Munter, T. R.; Moses, P. G.; Skúlason, E.; Bligaard, T.; Nørskov, J. K. Scaling Properties of Adsorption Energies for Hydrogen-Containing Molecules on Transition-Metal Surfaces. *Phys. Rev. Lett.* **2007**, *99*, 016105.

(7) Wang, S. et al. Universal transition state scaling relations for (de)hydrogenation over transition metals. *Phys. Chem. Chem. Phys.* **2011**, *13*, 20760–20765.

(8) Butler, J. A. V. Studies in heterogeneous equilibria. Part II.—The kinetic interpretation of the nernst theory of electromotive force. *Trans. Faraday Soc.* **1924**, *19*, 729–733.

(9) Erdey-Grúz, T.; Volmer, M. Zur Theorie der Wasserstoff Überspannung. *Zeitschrift für Physikalische Chemie* **1930**, *150A*, 203–213.

(10) Jinnouchi, R.; Kodama, K.; Nagoya, A.; Morimoto, Y. Simulated Volcano Plot of Oxygen Reduction Reaction on Stepped Pt Surfaces. *Electrochimica Acta* **2017**, *230*, 470–478.

(11) Stamenkovic, V.; Mun, B. S.; Mayrhofer, K. J.; Ross, P. N.; Markovic, N. M.; Rossmeisl, J.; Greeley, J.; Nørskov, J. K. Changing the Activity of Electrocatalysts for Oxygen Reduction by Tuning the Surface Electronic Structure. *Angewandte Chemie International Edition* **2006**, *45*, 2897–2901.

(12) Greeley, J.; Stephens, I. E. L.; Bondarenko, A. S.; Johansson, T. P.; Hansen, H. A.; Jaramillo, T. F.; Rossmeisl, J.; Chorkendorff, I.; Nørskov, J. K. Alloys of platinum and early transition metals as oxygen reduction electrocatalysts. *Nature Chemistry* **2009**, *1*, 552–556.

(13) Stephens, I. E. L.; Bondarenko, A. S.; Grønbjerg, U.; Rossmeisl, J.; Chorkendorff, I. Understanding the electrocatalysis of oxygen reduction on platinum and its alloys. *Energy Environ. Sci.* **2012**, *5*, 6744–6762.

(14) Stamenkovic, V. R.; Fowler, B.; Mun, B. S.; Wang, G.; Ross, P. N.; Lucas, C. A.; Marković, N. M. Improved Oxygen Reduction Activity on Pt<sub>3</sub>Ni(111) via Increased Surface Site Availability. *Science* **2007**, *315*, 493–497.

(15) Stephens, I. E. L.; Bondarenko, A. S.; Perez-Alonso, F. J.; Calle-Vallejo, F.; Bech, L.; Johansson, T. P.; Jepsen, A. K.; Frydendal, R.; Knudsen, B. P.; Rossmeisl, J.; Chorkendorff, I. Tuning the Activity of Pt(111) for Oxygen Electroreduction by Subsurface Alloying. *Journal of the American Chemical Society* **2011**, *133*, 5485–5491.

(16) Wada, N.; Nakamura, M.; Hoshi, N. Structural Effects on the Oxygen Reduction Reaction on Pt Single-Crystal Electrodes Modified with Melamine. *Electrocatalysis* **2020**, *11*, 275–281.

(17) Daimon, H.; Yamazaki, S.-i.; Asahi, M.; Ioroi, T.; Inaba, M. A Strategy for Drastic Improvement in the Durability of Pt/C and PtCo/C Alloy Catalysts for the Oxygen Reduction Reaction by Melamine Surface Modification. *ACS Catalysis* **2022**, *12*, 8976–8985.

(18) Behler, J.; Parrinello, M. Generalized Neural-Network Representation of High-Dimensional Potential-Energy Surfaces. *Phys. Rev. Lett.* **2007**, *98*, 146401.

(19) Bartók, A. P.; Payne, M. C.; Kondor, R.; Csányi, G. Gaussian Approximation Potentials: The Accuracy of Quantum Mechanics, without the Electrons. *Phys. Rev. Lett.* **2010**, *104*, 136403.

(20) Jinnouchi, R.; Karsai, F.; Verdi, C.; Asahi, R.; Kresse, G. Descriptors representing two- and three-body atomic distributions and their effects on the accuracy of machine-learned inter-atomic potentials. *The Journal of Chemical Physics* **2020**, *152*, 234102.

(21) Jinnouchi, R.; Karsai, F.; Kresse, G. On-the-fly machine learning force field generation: Application to melting points. *Phys. Rev. B* **2019**, *100*, 014105.

(22) Bartók, A. P.; Kondor, R.; Csányi, G. On representing chemical environments. *Phys. Rev. B* **2013**, *87*, 184115.

(23) Miwa, K.; Ohno, H. Molecular dynamics study on $\beta$-phase vanadium monohydride with machine learning potential. *Phys. Rev. B* **2016**, *94*, 184109.

(24) Jinnouchi, R.; Lahnsteiner, J.; Karsai, F.; Kresse, G.; Bokdam, M. Phase Transitions of Hybrid Perovskites Simulated by Machine-Learning Force Fields Trained on the Fly with Bayesian Inference. *Phys. Rev. Lett.* **2019**, *122*, 225701.

(25) Jinnouchi, R.; Karsai, F.; Kresse, G. Making free-energy calculations routine: Combining first principles with machine learning. *Phys. Rev. B* **2020**, *101*, 060201.

(26) Jinnouchi, R.; Miwa, K.; Karsai, F.; Kresse, G.; Asahi, R. On-the-Fly Active Learning of Interatomic Potentials for Large-Scale Atomistic Simulations. *The Journal of Physical Chemistry Letters* **2020**, *11*, 6946–6955.

(27) Jinnouchi, R.; Karsai, F.; Verdi, C.; Kresse, G. First-principles hydration free energies of oxygenated species at water–platinum interfaces. *The Journal of Chemical Physics* **2021**, *154*, 094107.

(28) Jinnouchi, R. Molecular dynamics simulations of proton conducting media containing phosphoric acid. *Phys. Chem. Chem. Phys.* **2022**, *24*, 15522–15531.

(29) Jinnouchi, R.; Minami, S.; Karsai, F.; Verdi, C.; Kresse, G. Proton Transport in Perfluorinated Ionomer Simulated by Machine-Learned Interatomic Potential. *The Journal of Physical Chemistry Letters* **2023**, *14*, 3581–3588.

(30) Minami, S.; Jinnouchi, R. Accelerating anhydrous proton conduction via anion rotation and hydrogen bond recombination: a machine-learning molecular dynamics. *J. Mater. Chem. A* **2023**, *11*, 16104–16114.

(31) Jinnouchi, R.; Karsai, F.; Kresse, G. Machine learning-aided first-principles calculations of redox potentials. *npj Computational Materials* **2024**, *10*, 107.

(32) Kresse, G.; Furthmüller, J. Efficient iterative schemes for ab initio total-energy calculations using a plane-wave basis set. *Phys. Rev. B* **1996**, *54*, 11169.

(33) Kresse, G.; Furthmüller, J. Efficiency of ab-initio total energy calculations for metals and semiconductors using a plane-wave basis set. *Computational Materials Science* **1996**, *6*, 15.

(34) Hammer, B.; Hansen, L. B.; Nørskov, J. K. Improved adsorption energetics within density-functional theory using revised Perdew-Burke-Ernzerhof functionals. *Phys. Rev. B* **1999**, *59*, 7413–7421.

(35) Grimme, S.; Antony, J.; Ehrlich, S.; Krieg, H. A consistent and accurate ab initio parametrization of density functional dispersion correction (DFT − D) for the 94 elements H-Pu. *The Journal of Chemical Physics* **2010**, *132*, 154104.

(36) Grimme, S. Density functional theory with London dispersion corrections. *WIREs Computational Molecular Science* **2011**, *1*, 211–228.

(37) Kresse, G.; Joubert, D. From ultrasoft pseudopotentials to the projector augmented-wave method. *Phys. Rev. B* **1999**, *59*, 1758–1775.

(38) Jinnouchi, R. Grand-Canonical First Principles-Based Calculations of Electrochemical Reactions. *Journal of The Electrochemical Society* **2024**, *171*, 096502.

(39) Nosé, S. A unified formulation of the constant temperature molecular dynamics methods. *The Journal of Chemical Physics* **1984**, *81*, 511–519.

(40) Hoover, W. G. Canonical dynamics: Equilibrium phase-space distributions. *Phys. Rev. A* **1985**, *31*, 1695–1697.

(41) Jinnouchi, R.; Karsai, F.; Kresse, G. Absolute standard hydrogen electrode potential and redox potentials of atoms and molecules: machine learning aided first principles calculations. *arXiv* **2024**,

(42) Jinnouchi, R. Machine-learning surrogate models for particle insertions and element substitutions. *The Journal of Chemical Physics* **2024**, *161*, 194110.

(43) Ambrosio, F.; Miceli, G.; Pasquarello, A. Redox levels in aqueous solution: Effect of van der Waals interactions and hybrid functionals. *The Journal of Chemical Physics* **2015**, *143*, 244508.

(44) Ambrosio, F.; Guo, Z.; Pasquarello, A. Absolute Energy Levels of Liquid Water. *The Journal of Physical Chemistry Letters* **2018**, *9*, 3212–3216.

(45) Tada, K.; Yamazaki, S.-i.; Asahi, M.; Ioroi, T. Elucidation of the mechanism of melamine adsorption on Pt(111) surface via density functional theory calculations. *Phys. Chem. Chem. Phys.* **2023**, *25*, 23047–23057.

(46) Sun, H. Force field for computation of conformational energies, structures, and vibrational frequencies of aromatic polyesters. *Journal of Computational Chemistry* **1994**, *15*, 752–768.

(47) Le, J.; Iannuzzi, M.; Cuesta, A.; Cheng, J. Determining Potentials of Zero Charge of Metal Electrodes versus the Standard Hydrogen Electrode from Density-Functional-Theory-Based Molecular Dynamics. *Phys. Rev. Lett.* **2017**, *119*, 016801.

(48) Trasatti, S. The absolute electrode potential: an explanatory note (Recommendations 1986). *Pure and Applied Chemistry* **1986**, *58*, 955–966.

(49) Xu, P.; von Rueden, A. D.; Schimmenti, R.; Mavrikakis, M.; Suntivich, J. Optical method for quantifying the potential of zero charge at the platinum–water electrochemical interface. *Nature Materials* **2023**, *22*, 503–510.

(50) Nørskov, J. K.; Bligaard, T.; Logadottir, A.; Kitchin, J. R.; Chen, J. G.; Pandelov, S.; Stimming, U. Trends in the Exchange Current for Hydrogen Evolution. *Journal of The Electrochemical Society* **2005**, *152*, J23.

(51) Greeley, J.; Jaramillo, T. F.; Bonde, J.; Chorkendorff, I.; Nørskov, J. K. Computational high-throughput screening of electrocatalytic materials for hydrogen evolution. *Nature Materials* **2006**, *5*, 909–913.

(52) Man, I. C.; Su, H.-Y.; Calle-Vallejo, F.; Hansen, H. A.; Martínez, J. I.; Inoglu, N. G.; Kitchin, J.; Jaramillo, T. F.; Nørskov, J. K.; Rossmeisl, J. Universality in Oxygen Evolution Electrocatalysis on Oxide Surfaces. *ChemCatChem* **2011**, *3*, 1159–1165.

(53) Seitz, L. C.; Dickens, C. F.; Nishio, K.; Hikita, Y.; Montoya, J.; Doyle, A.; Kirk, C.; Vojvodic, A.; Hwang, H. Y.; Norskov, J. K.; Jaramillo, T. F. A highly active and stable IrO<i><sub>x</sub></i>/SrIrO<sub>3</sub> catalyst for the oxygen evolution reaction. *Science* **2016**, *353*, 1011–1014.

(54) Peterson, A. A.; Nørskov, J. K. Activity Descriptors for CO2 Electroreduction to Methane on Transition-Metal Catalysts. *The Journal of Physical Chemistry Letters* **2012**, *3*, 251–258.

(55) Liu, X.; Xiao, J.; Peng, H.; Hong, X.; Chan, K.; Nørskov, J. K. Understanding trends in electrochemical carbon dioxide reduction rates. *Nature Communications* **2017**, *8*, 15438.

(56) Nitopi, S.; Bertheussen, E.; Scott, S. B.; Liu, X.; Engstfeld, A. K.; Horch, S.; Seger, B.; Stephens, I. E. L.; Chan, K.; Hahn, C.; Nørskov, J. K.; Jaramillo, T. F.; Chorkendorff, I. Progress and Perspectives of Electrochemical CO2 Reduction on Copper in Aqueous Electrolyte. *Chemical Reviews* **2019**, *119*, 7610–7672.

(57) Montoya, J. H.; Tsai, C.; Vojvodic, A.; Nørskov, J. K. The Challenge of Electrochemical Ammonia Synthesis: A New Perspective on the Role of Nitrogen Scaling Relations. *ChemSusChem* **2015**, *8*, 2180–2186.

(58) Qing, G.; Ghazfar, R.; Jackowski, S. T.; Habibzadeh, F.; Ashtiani, M. M.; Chen, C.-P.; Smith III, M. R.; Hamann, T. W. Recent Advances and Challenges of Electrocatalytic N2 Reduction to Ammonia. *Chemical Reviews* **2020**, *120*, 5437–5516.
